# *Sprouty* genes regulate activated fibroblasts in mammary epithelial development and breast cancer

**DOI:** 10.1038/s41419-024-06637-2

**Published:** 2024-04-10

**Authors:** Jiyong Li, Rongze Ma, Xuebing Wang, Yunzhe Lu, Jing Chen, Deyi Feng, Jiecan Zhou, Kun Xia, Ophir Klein, Hao Xie, Pengfei Lu

**Affiliations:** 1 MOE Key Lab of Rare Pediatric Diseases & Hunan Key Laboratory of Medical Genetics of the School of Life Sciences, Hu Nan Sheng, China; 2 Institute of Cell Biology, University of South China, Hu Nan Sheng, China; 3 Institute for Future Sciences, Hengyang Medical School, University of South China, Hu Nan Sheng, China; 4 Institute of Aix-Marseille, Wuhan University of Technology, Wuhan, 430070 China; 5 School of Chemistry, Chemical Engineering and Life Sciences, Wuhan University of Technology, Wuhan, 430070 China; 6 School of Life Science and Technology, ShanghaiTech University, Shanghai, 201210 China; 7 The First Affiliated Hospital, Pharmacy Department, Hengyang Medical School, University of South China, Hu Nan Sheng, China; 8 Department of Orofacial Sciences and Program in Craniofacial Biology, University of California, San Francisco, UCSF Box 0422, 513 Parnassus Avenue, HSE1508, San Francisco, CA 94143 California, USA; 9 Department of Pediatrics and Guerin Children’s, Cedars-Sinai Medical Center, 8700 Gracie Allen Dr. Los Angeles, CA USA

**Keywords:** Morphogenesis, Breast cancer

## Abstract

Stromal fibroblasts are a major stem cell niche component essential for organ formation and cancer development. Fibroblast heterogeneity, as revealed by recent advances in single-cell techniques, has raised important questions about the origin, differentiation, and function of fibroblast subtypes. In this study, we show in mammary stromal fibroblasts that loss of the receptor tyrosine kinase (RTK) negative feedback regulators encoded by *Spry1*, *Spry2*, and *Spry4* causes upregulation of signaling in multiple RTK pathways and increased extracellular matrix remodeling, resulting in accelerated epithelial branching. Single-cell transcriptomic analysis demonstrated that increased production of FGF10 due to *Sprouty* (*Spry*) loss results from expansion of a functionally distinct subgroup of fibroblasts with the most potent branching-promoting ability. Compared to their three independent lineage precursors, fibroblasts in this subgroup are “activated,” as they are located immediately adjacent to the epithelium that is actively undergoing branching and invasion. *Spry* genes are downregulated, and activated fibroblasts are expanded, in all three of the major human breast cancer subtypes. Together, our data highlight the regulation of a functional subtype of mammary fibroblasts by *Spry* genes and their essential role in epithelial morphogenesis and cancer development.

## Introduction

The stromal microenvironment, composed of immune cells, fibroblasts, endothelial cells, and their non-cellular products, including cytokines, growth factors, and extracellular matrix (ECM), is essential for stem cell biology during epithelial organ formation, homeostasis, and cancer development [1–3]. Recent successes in immunotherapy have raised great hope that targeting cancer associated fibroblasts (CAFs), which are essential for cancer development, may bring about another revolution in cancer therapeutics [4, 5]. Like immune cells, fibroblasts are a heterogeneous population and, based on their different molecular make-ups, fibroblast subgroups may perform distinct functions in development and cancer [6]. At present, a major challenge in targeting CAFs is to understand the function of distinct fibroblast subtypes, as revealed by single-cell biology in organ development and homeostasis, and how they are transformed into CAFs to promote cancer initiation and progression [7, 8].

The mouse mammary gland provides an experimentally tractable system to understand the biology of normal and cancer associated fibroblasts [9]. Interactions between epithelium and mesenchyme, or its postnatal derivative the stromal fibroblasts are essential for mammary development and breast cancer [10]. Our work and others have shown that signaling via receptor-tyrosine kinases (RTKs) constitutes an interaction loop between mammary epithelium and stromal fibroblasts [11, 12]. On the one hand, mammary fibroblasts are a rich source of RTK ligands, including those that bind to the fibroblast growth factor receptor (FGFR), insulin growth factor receptor (IGFR), and vascular growth factor receptor (VGFR) families. A reduction in ligand production by stromal fibroblasts often leads to a decrease in stromal-to-epithelial RTK signaling and stunted branching [13–16]. By contrast, overactive epithelial RTK signaling, due to excessive RTK ligand production or RTK activities, causes breast tumorigenesis in mouse models [17–19].

On the other hand, RTKs also mediate epithelial-to-stromal signaling and regulate mammary gland branching. For example, epithelial-derived amphiregulin (AREG) and transforming growth factor alpha (TGFα), both of which are ligands of the ERBB family member EGF receptor and are required for epithelial morphogenesis; in their absence, EGFR signaling is reduced, leading to reduced mammary gland branching [20]. On the contrary, excessive EGFR signaling causes breast tumorigenesis and is a therapeutic target of human cancer [21, 22]. We previously showed that *Spry1*, a member of the *Sprouty* (*Spry*) family [23–25], modulates epithelial–stromal interactions by inhibiting EGFR-dependent stromal paracrine signaling and ECM remodeling [12]. In the current study, we examined the function of other members of the *Spry* family by removing all the three members from mammary stromal fibroblasts.

## Results

### Loss of *Spry1*, *Spry2*, and *Spry4* in mammary stromal fibroblasts causes accelerated epithelial branching

**Fig. 1:**
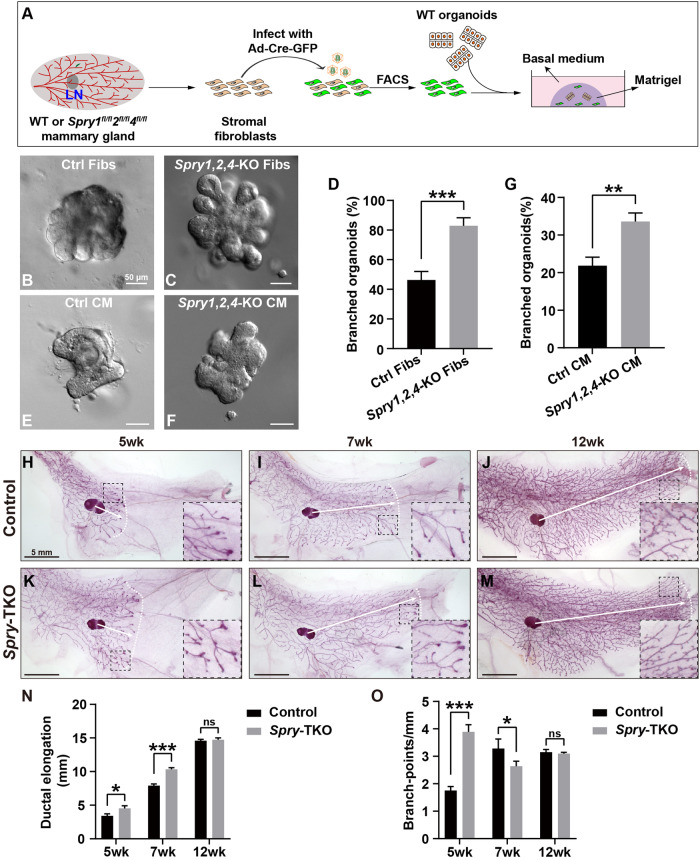
Loss of *Spry1*, *Spry2*, and *Spry4* in mammary stromal fibroblasts causes accelerated epithelial branching. **A** Schematic diagram depicting the experimental procedures during sample preparation, in vitro culture, and treatment methods. Mammary organoids and stromal fibroblasts were prepared from wild type (WT) and *Spry1*^fl/fl^; *Spry2*^fl/fl^; *Spry4*^fl/fl^ mice. Fibroblasts with and without *Spry1*, *Spry2*, and *Spry4* function were acquired by infection with Ad-Cre-GFP and FACS-mediated selection of GFP-positive infected cells. Wild type organoids were co-cultured with either control fibroblasts or *Spry1*,*2*,*4*-KO fibroblasts, both of which embedded in Matrigel. Abbreviation: LN, lymph node. **B**–**D** Wildtype organoids co-cultured with control (**B**) and *Spry1*,*2*,*4*-KO fibroblasts (**C**). **D** Quantification of the number of organoid branches co-cultured with fibroblasts (Fibs). **E**–**G** Wildtype organoids cultured using conditioned medium (CM) from control (**E**) or *Spry1*,*2*,*4*-TKO fibroblasts (**F**). **G** Quantification of the number of organoid branches cultured using medium from control or *Spry1*,*2*,*4*-KO fibroblasts. Data were from three independent experiments and were presented as mean ± SD. **P < 0.01; ***P < 0.001. Scale bars: 50 μm. **H**–**O** Branching trees of the #4 mammary glands, as revealed by Carmine Red staining of control (**H**–**J**) and mutant *Spry*-TKO (**K**–**M**) whole-mount glands, at the 5-wk, 7-wk, and 12-wk stages. Female mice in each comparison group were from the same litter and were checked to ensure that they were in the same estrous state before sacrifice. Arrows indicate the extent of ductal penetration in the fat pad. The dotted white line illustrates the epithelial invasion front. Insets show closer view of the area in a square with black dotted line. **N**, **O** Quantitative comparisons of ductal penetration and branching points per millimeter between control and mutant glands. Plots show mean ± SD (n $\ge$3/genotype); ns, not significant; *P < 0.05; ***P < 0.001; Scale bars: 5 mm.

Using quantitative real-time PCR (qPCR), we found that *Spry1*, *Spry2*, and *Spry4* of the *Spry* family were expressed in the stromal fibroblasts of the 7-week-old mammary gland (Supplementary Fig. 1A). To determine the knockout efficiency of *Spry1*, *Spry2*, and *Spry4*, we harvested mammary stromal fibroblasts from wild type and *Spry1*^fl/fl^; *Spry2*^fl/fl^; *Spry4*^fl/fl^ mice and infected them with Adenovirus (Ad)-Cre-GFP to generate control (*Spry1*^+/+^; *Spry2*^+/+^; *Spry4*^+/+^) and mutant (*Spry1*^∆/∆^; *Spry2*^∆/∆^; *Spry4*^∆/∆^, or *Spry1*,*2*,*4*-KO) fibroblasts, respectively (Fig. 1A). We found that Ad-Cre-mediated knockout efficiently removed mRNA expression of all three *Spry* genes (Supplementary Fig. 1B). We then harvested wild-type epithelial organoids and cultured them together either with control or mutant fibroblasts. We found that, after a four-day culture, ~42% organoids co-cultured with control fibroblasts formed branches (Fig. 1B, D), whereas ~80% organoids did so when co-cultured with mutant fibroblasts and their branches were more pronounced (Fig. 1C, D).

Next, we used conditioned medium from either control or mutant fibroblasts to culture wild-type organoids. We found there was a considerable drop in branching efficiency, as organoids cultured in control conditioned medium were able to form branches in ~20% cases and the branches were not as prominent as when co-cultured with fibroblasts (Fig. 1E, G). A similar drop in branching efficiency was also observed when mutant fibroblasts were replaced with mutant conditioned medium (Fig. 1C, F, G). However, organoids co-cultured with mutant conditioned medium formed at a higher percentage ( ~ 35%) than with wild-type conditioned medium. The results suggest that *Spry* loss in mammary fibroblasts promotes mammary epithelial branching, and this is, at least in part, via paracrine factors.

We then sought to examine whether loss of *Spry1*, *Spry2*, and *Spry4* causes overgrowth of the mammary gland in vivo. We crossed male mice hemizygous for the *Fsp1-cre* transgene [26] and heterozygous for the *Spry1*^∆^; *Spry2*^∆^; *Spry4*^∆^ alleles with female mice homozygous for the *Spry1*^fl/fl^; *Spry2*^fl/fl^; *Spry4*^fl/fl^ alleles. All *Fsp1-cre*; *Spry1*^Δ/fl^; *Spry2*^Δ/fl^; *Spry4*^Δ/fl^ progeny (*Spry* triple knockout, or *Spry-*TKO) were viable and were used to compare with their control littermates *Fsp1-cre*; *Spry1*^fl/+^; *Spry2*^fl/+^; *Spry4*^fl/+^ (Control). Using the lymph node in the proximal mammary fat-pad as a landmark, we examined mammary epithelial development based on the lengths of ductal elongation and the density of branch-points at the 5-week, 7-week, and 12-week stages. As expected, we saw progressive ductal elongation from 5-week to 12-week in the control mammary gland epithelium (Fig. 1H–J, N). Ductal elongation was significantly accelerated in the *Spry*-TKO at both the 5-week and 7-week stages compared with those in the control glands (Fig. 1H–M, N). However, an increase in branch-point formation was only observed at the 5-week stage in the mutant glands (Fig. 1H–M, O). By 12-weeks, the wild-type had caught up in terms of ductal elongation, and the number of branch-points formed regressed in the mutant, such that no significant differences were observed between *Spry*-TKO and control glands (Fig. 1H–O).

Together, the data show that *Spry1*, *Spry2*, and *Spry4* are expressed in the mammary gland stromal fibroblasts and inhibit epithelial branching, at least in part, by regulating the production of fibroblast paracrine factors essential for branching.

### Multiple RTK signaling pathways in the fibroblasts are sensitized due to the loss of *Spry1*, *Spry2*, and *Spry4*

To determine how the loss of *Spry* genes affects RTK signaling in the stromal fibroblasts, we performed mass spectrometry on phosphoproteins using both control and mutant fibroblasts lacking *Spry1*, *Spry2*, and *Spry4* functions (Supplementary Fig. 2A). We found that signaling activity via the ERBB family, consisting of EGFR, ERBB2, ERBB3, and ERBB4, was up-regulated in *Spry1*,*2*,*4*-KO fibroblasts when compared to control fibroblasts (Supplementary Fig. 2B). Moreover, signaling via the VEGF receptors, including FLT1, KDR, and FLT4, and the insulin growth factor receptors, composed of IGF1R and IGF2R, was also increased in the mutant fibroblasts when compared to control fibroblasts (Supplementary Fig. 2B).

To confirm the mass spectrometry results, we stimulated both *Spry1*,*2*,*4*-KO fibroblasts and control fibroblasts with VEGF and EGF, which are ligands of the VEGFR and ERBB families, respectively, and measured ERK phosphorylation at the Thr202/Tyr204 sites as an indicator of the levels of signaling activation at different durations of ligand stimulation. We also used fetal bovine serum (FBS), which contains various growth factors including RTK ligands, to treat mutant and control fibroblasts under similar experimental conditions as EGF and VEGF ligands. We found that ERK phosphorylation at Thr202/Tyr204 was upregulated in *Spry1*,*2*,*4*-KO fibroblasts when compared with control fibroblasts when stimulated by FBS (Supplementary Fig. 2C, D), VEGF (Supplementary Fig. 2E, F) and EGF (Supplementary Fig. 2G, H).

The results thus show that when *Spry1*, *Spry2*, and *Spry4* were deleted from mammary fibroblasts, signaling activities of multiple RTK pathways were increased, suggesting that *Spry1*, *Spry2*, and *Spry4* negatively regulate these pathways during normal mammary gland development.

### Loss of *Spry* genes in mammary fibroblasts leads to increased FGF10 but decreased FGF2 signaling in the epithelium

**Fig. 2:**
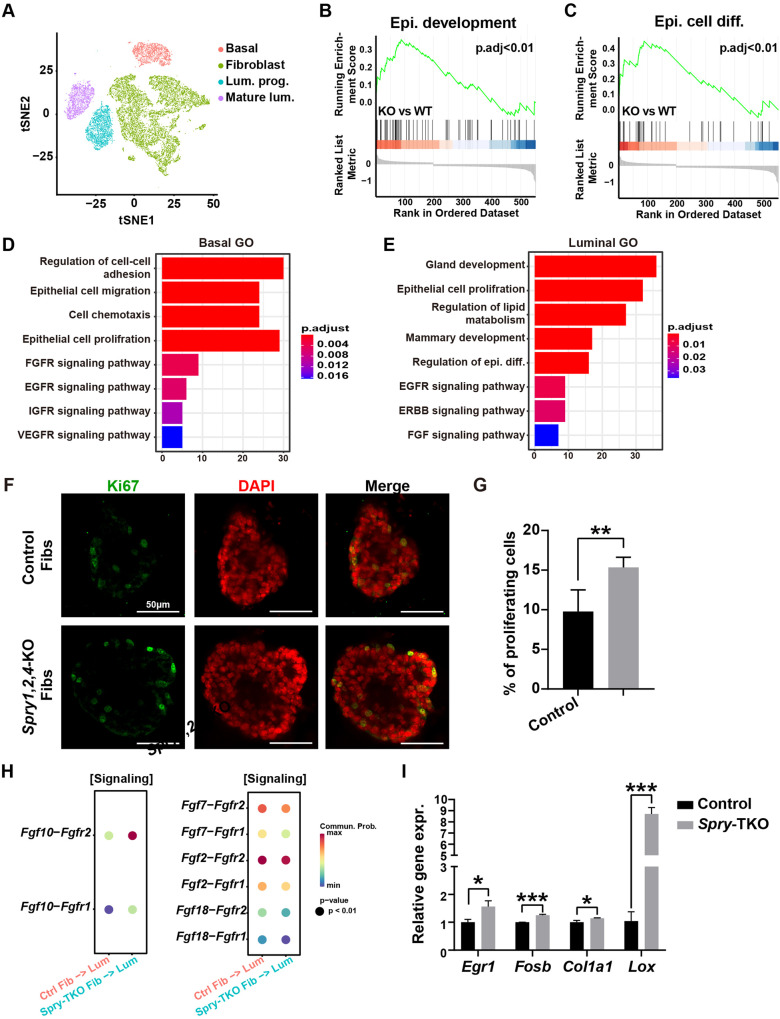
Loss of *Spry* genes in mammary fibroblasts leads to increased FGF10 but decreased FGF2 signaling in the epithelium. **A** t-SNE plot showing the combined single cell transcriptomes of mammary epithelial cells and stromal fibroblasts isolated from seven-week-old control and *Spry-*TKO mammary glands (*n* = 2 female mice). Abbreviations: Lum, luminal; prog, progenitor. **B**, **C** GSEA analysis of pathways related to epithelial development and cell differentiation in control and *Spry-*TKO mammary glands. Abbreviations: diff., differentiation; epi., epithelial; KO, knockout; WT, wildtype. **D**, **E** GO analysis of the main pathway changes in basal cells (**D**) and luminal cells (**E**) of control and *Spry-*TKO mammary glands. **F**, **G** Cell proliferation of wild-type organoid epithelium co-cultured with control and *Spry1*,*2*,*4*-KO fibroblasts (Fibs) as detected by Ki67 staining (**F**) and was quantified (**G**). Data were from three independent experiments and were presented as mean ± SD. **P < 0.01. Scale bars: 50 μm. **H** Cellchat analysis of either increased or decreased fibroblast-to-epithelial FGF ligand-receptor signaling in *Spry-*TKO mammary glands when compared with control glands. Abbreviations: ctrl., control; fib., fibroblasts; lum., luminal. **I** qPCR analysis of target genes in the FGF signaling pathway. Abbreviation: Expr, expression. *P < 0.05; ***P < 0.001.

Next, we wanted to take advantage of single cell transcriptomics to examine the consequence of *Spry* loss in the stroma on both epithelial cell behavior and fibroblast biology at the molecular level. Thus, we harvested epithelial and fibroblast cells from control glands and *Spry-*TKO glands at the 7-week stage and performed single cell RNA sequencing (scRNA-seq). Using the t-SNE plot analysis, we found that mammary cells were separated into fibroblasts, and three subtypes of epithelial cells, including basal cells, luminal progenitors, and mature luminal cells (Fig. 2A).

We first focused on transcriptomic changes in the epithelial cells due to loss of *Spry1*, *Spry2*, and *Spry4* in the fibroblasts. Consistent with an accelerated branching phenotype, Gene Set Enrichment Analysis (GSEA) showed increased epithelial development and differentiation in mammary epithelial cells from *Spry*-TKO mice when compared with control epithelial cells (Fig. 2B, C). Gene ontology (GO) analyses showed that cell proliferation, differentiation, communication, and signal transduction pathways, among others, were increased in basal and luminal cells of the mutant glands (Fig. 2D, E). The data thus indicated that epithelial cells upregulate behavior essential for branching morphogenesis because of loss of *Spry* genes in fibroblasts.

To validate the scRNA transcriptomic data showing increased epithelial development in the mutant gland, we examined the expression of Ki67, a mitotic marker, by immunofluorescence in organoid epithelium co-cultured either with control fibroblasts or *Spry-*TKO fibroblasts. We found that epithelial cell proliferation increased by ~50% when organoids were co-cultured with *Spry-*TKO fibroblasts rather than control fibroblasts (Fig. 2F, G).

Next, we explored the scRNA-seq dataset and examined the activities of FGF-FGFR signaling, which is the best characterized pathway mediating stroma-to-epithelium signaling during mammary epithelial branching [16, 25]. Interestingly, we found FGF10-FGFR1/2 signaling was increased, whereas FGF2-, FGF7-, and FGF18-FGFR1/2 signaling was decreased in the epithelium of the *Spry-*TKO glands when compared with control epithelium (Fig. 2H). Considering that FGF10 is the most dominantly expressed and the most bioactive stromal FGF ligand essential for epithelial morphogenesis as we previously reported [25, 27, 28], we predicted that the overall FGF signaling would have been increased in the epithelium of *Spry-*TKO glands. Using qPCR, we examined mRNA expression of the FGF signaling target genes *Egr1* and *Fosb*, and the ECM remodeling enzyme *Lox* in the epithelial cells [25, 28]. Consistent with the scRNA-seq data, we found that the expression of these FGF signaling target genes was increased in the mutant gland epithelium (Fig. 2I).

Together, the data show that the loss of *Spry* genes in the mammary stromal fibroblasts leads to an increase in FGF signaling, cell proliferation and invasion in the epithelium, ultimately leading to accelerated branching morphogenesis. Notably, while the overall FGF signaling level increases in the epithelium, only FGF10 signaling exhibits an increase, whereas FGF2, FGF7, and FGF18 signaling decrease. These findings suggest that the production of these ligands in the fibroblasts is increased or decreased, respectively.

### *Spry* genes regulate the number of fibroblasts that express *Fgf* ligands

**Fig. 3:**
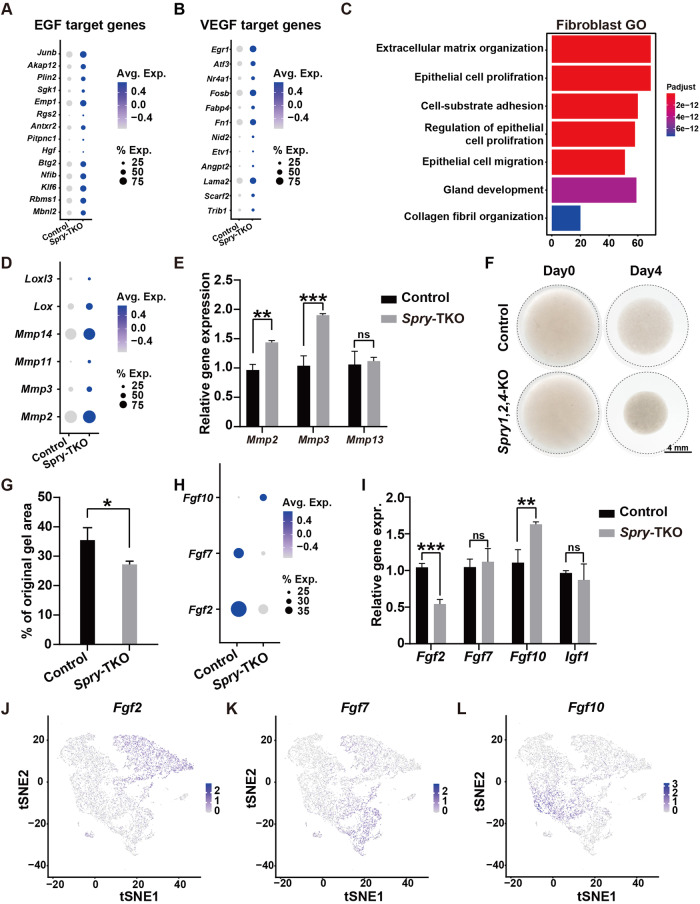
*Spry* genes regulate the number of fibroblasts that express *Fgf* ligands. **A**, **B** Comparison of transcriptional expression of target genes in the EGF (**A**) and VEGF (**B**) signaling pathways based on the scRNA-seq data from control and *Spry-*TKO glands. Note that changes of color intensity indicate the levels of increased or decreased mRNA expression, whereas the area of each circle correlates with the percentage of fibroblasts that express the target gene of interest. Also note that, although the average expression of these genes was changed in the mutant glands, the percentage of the fibroblasts expressing each gene remained like the control glands. **C** GO analysis of the main pathway changes in fibroblasts of control and *Spry-*TKO mammary glands. **D**, **E** Comparison of transcriptional expression of several key ECM remodeling enzymes based on scRNA-seq data (**D**). Validation of the gene expression changes, using several *Mmp*s as an example, by qPCR experiments (**E**). **F**, **G** Collagen contraction assay. **F** Photographs of a representative experiment. **G** Graph shows size change of collagen gels, expressed as a percentage of the original area ± SD (*n* = 3). *P < 0.05. **H**, **I** Comparison of transcriptional expression of *Fgf2*, *Fgf7*, and *Fgf10* based on the scRNA-seq data (**H**). Note that both the average expression levels and the percentages of fibroblasts expressing these *Fgf* genes were changed in the *Spry-*TKO fibroblasts. Specifically, unlike in (**A**, **B**), the percentages of cells that express the *Fgf* genes changed in mutant fibroblasts. Validation of the mRNA expression changes of the *Fgf* ligands by qPCR experiments (**I**). **J**–**L** t-SNE plots showing the combined single cell transcriptomes of mammary stromal fibroblasts with regards to *Fgf2* (**J**), *Fgf7* (**K**), and *Fgf10* expression (**L**). Note that these *Fgfs* are expressed by distinct, though overlapping clusters of fibroblasts. Abbreviations: Avg. average; Exp. Expression. Data were from three independent experiments and were presented as mean ± SD. ns, not significant; *P < 0.05; **P < 0.01; ***P < 0.001.

We then shifted our focus to stromal fibroblasts with the aim of finding the molecular events underlying increased epithelial branching in *Spry-*TKO mammary glands. We first examined the transcriptional expression of *Cbl* and *Tgfb1*, both of which function similarly as *Sprouty* genes in the mammary gland stromal fibroblasts to inhibit epithelial branching [29, 30]. However, we did not detect a difference in their expression in any of the MSF subgroups (Supplementary Fig. 3A, B). However, consistent with increased EGF and VEGF signaling from the mass spectrometry phosphoprotein data (Supplementary Fig. 2), we found that the target genes of both signaling pathways were up-regulated at the mRNA level in the *Spry*-TKO fibroblasts when compared to control fibroblasts (Fig. 3A, B) [31–33]. Moreover, pathways regulating cell proliferation, migration, collagen organization, and ECM organization, among others, were also increased in their activities in the *Spry*-TKO fibroblasts based on both GO analysis (Fig. 3C) and mRNA expression of ECM remodeling enzymes, including *Lox*, *Loxl3*, *Mmp2*, and *Mmp14* (Fig. 3D). Using qPCR, we confirmed that mRNA expression of *Mmp2* and *Mmp3*, but not *Mmp13*, was significantly higher in *Spry*-TKO fibroblasts than in control fibroblasts (Fig. 3E). Using the collagen contraction assay, we directly examined the ECM remodeling activities of *Spry*-TKO fibroblasts and control fibroblasts. We found that collagen gels contracted by 65% in the presence of control fibroblasts after a four-day culture; by contrast, their contraction was stronger in the presence of *Spry*-TKO fibroblasts as, in this case, the gel shrunk by 73% (Fig. 3F, G), indicating that ECM remodeling activities were significantly higher in *Spry*-TKO fibroblasts than in control fibroblasts.

Next, we examined mRNA expression of several essential FGF ligands in the stromal fibroblasts. Consistent with the results showing the FGF ligand-receptor signaling activities in the epithelial cells (Fig. 2H), we found that the average stromal mRNA expression of *Fgf10* was higher than control in *Spry*-TKO fibroblasts; however, the average mRNA expression of *Fgf2* and *Fgf7* was lower than control *Spry*-TKO fibroblasts (Fig. 3H). Moreover, the data show that the number of *Fgf10*-expressing cells was larger than control, whereas the number of both *Fgf2* and *Fgf7* was smaller than control in *Spry*-TKO glands (Fig. 3H). Using qPCR, we confirmed that *Fgf2* and *Fgf10* expression levels were higher in control fibroblasts and *Spry*-TKO fibroblasts, respectively, whereas no significant changes were observed for *Fgf7* or *Igf1* (Fig. 3I).

Considering that stroma-to-epithelial FGF-FGFR signaling is overall increased in *Spry*-TKO glands (Fig. 2I), it is intriguing that *Spry* loss causes opposite effects on fibroblast production of *Fgf2* and *Fgf10*. Such a differential effect on *Fgf2* and *Fgf10* expression could result from changes in transcriptional regulation of each individual gene and/or in the number of fibroblasts that express these two genes. Therefore, we directly examined the cells that expressed *Fgf2*, *Fgf7*, and *Fgf10* using the UMAP plots. Strikingly, we found that levels of *Fgf2*, *Fgf7*, and *Fgf10* mRNA expression varied in fibroblast cells, and that they were enriched in different populations of mammary stromal fibroblasts (Fig. 3J–L).

Together, the data confirm that *Spry1*, *Spry2*, and *Spry4* inhibit VEGF and EGF signaling and negatively regulate ECM remodeling activities in mammary fibroblasts. *Spry* genes also regulate FGF ligand production, though interestingly by regulating the number of fibroblasts that express different FGF ligands.

### MSF-2 subgroup is likely derived from three independent fibroblast lineages

**Fig. 4:**
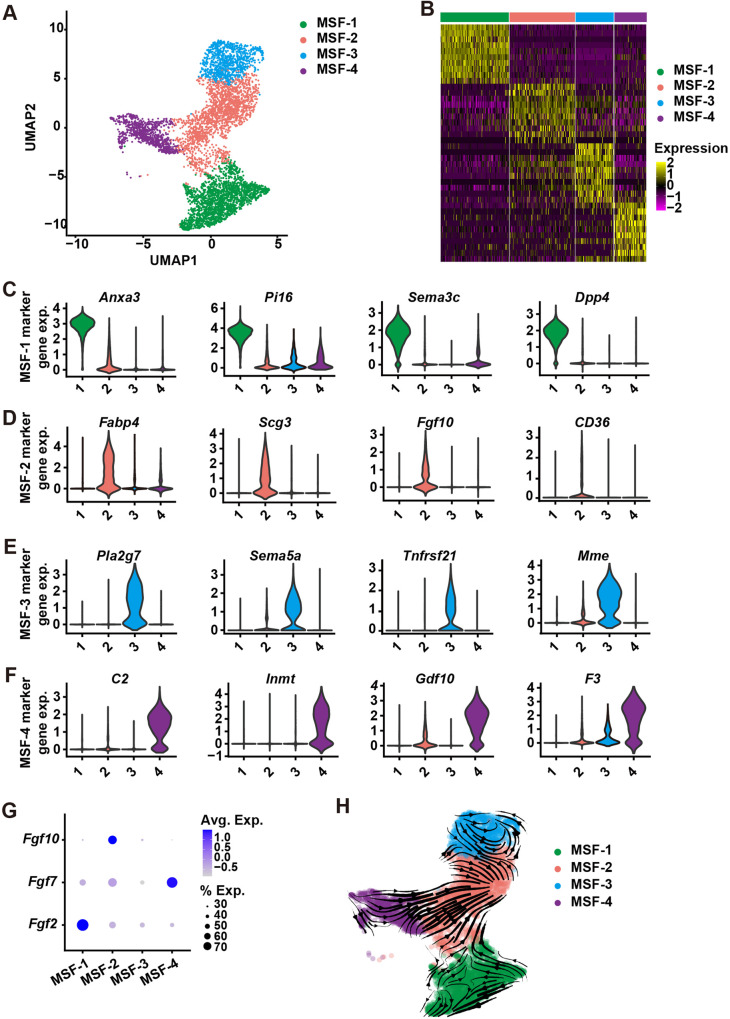
MSF-2 subgroup is derived from three independent fibroblast lineages. **A** t-SNE plot showing the single cell transcriptomics of mammary stromal fibroblasts from the control glands. **B** Heatmap showing relative log-expression of the top 10 marker genes for each cell cluster identified in Fig. 4A. Abbreviation: exp., expression. **C**–**F** mRNA expression of four representative genes from MSF-1 (**C**), MSF-2 (**D**), MSF-3 (**E**), and MSF-4 (**F**). **G** mRNA expression *Fgf2*, *Fgf7*, and *Fgf10* in the MSF1-4 subgroups. Avg. Average, Exp. Expression. **H** Velocity analysis showing multiple origins of the MSF-2 population. Note that arrows indicate the differentiation directions.

The above results imply that there are different subtypes of stromal fibroblasts in the mammary gland. Using UMAP analysis, we identified four distinct subpopulations of mammary stromal fibroblasts (MSFs), which we defined as MSF-1, MSF-2, MSF-3, and MSF-4 (Fig. 4A). Using the heatmap analysis, we discovered top10 most highly expressed genes that could be used as a panel of marker genes to define each of the MSF subpopulations (Fig. 4B and Supplementary Fig. 4A). For example, *Anxa3*, *Pi16*, *Sema3c*, and *Dpp4* were most highly expressed in MSF-1 (Fig. 4C), but not in other subpopulations. Likewise, *Fabp4*, *Scg3*, *Fgf10*, and *CD36* were specific for MSF-2 (Fig. 4D); *Pla2g7*, *Sem5a*, *Tnfrsf21*, and *Mme* were specific for MSF-3 (Fig. 4E); whereas *C2*, *Inmt*, *Gdf10*, and *F3* were specific for MSF-4 (Fig. 4F).

As expected, we found that *Fgf2*, *Fgf7*, and *Fgf10* were enriched in different fibroblast subpopulations, i.e., in MSF-1, MSF4, and MSF-2, respectively (Fig. 4G). To examine the differentiation trajectory of the MSFs, we performed the Velocity assay, which analyzes the ratio of un-spliced to spliced mRNA from scRNA-seq data and predicts future cell states based on the current transcriptional state [34]. Interestingly, we found that the three MSF populations, including the MSF-1, MSF-3, and MSF-4 populations, were developmentally independent and that they contributed to MSF-2 formation (Fig. 4H). Using the pseudotime analysis, we confirmed this conclusion (Supplementary Fig. 4B, C).

Together, the data confirm that different MSF subpopulations uniquely produce different combinations of FGF ligands and suggest that MSF-2 is derived from the three other independent MSF lineages.

### *Spry* genes inhibit expansion of MSF-2 subpopulation

**Fig. 5:**
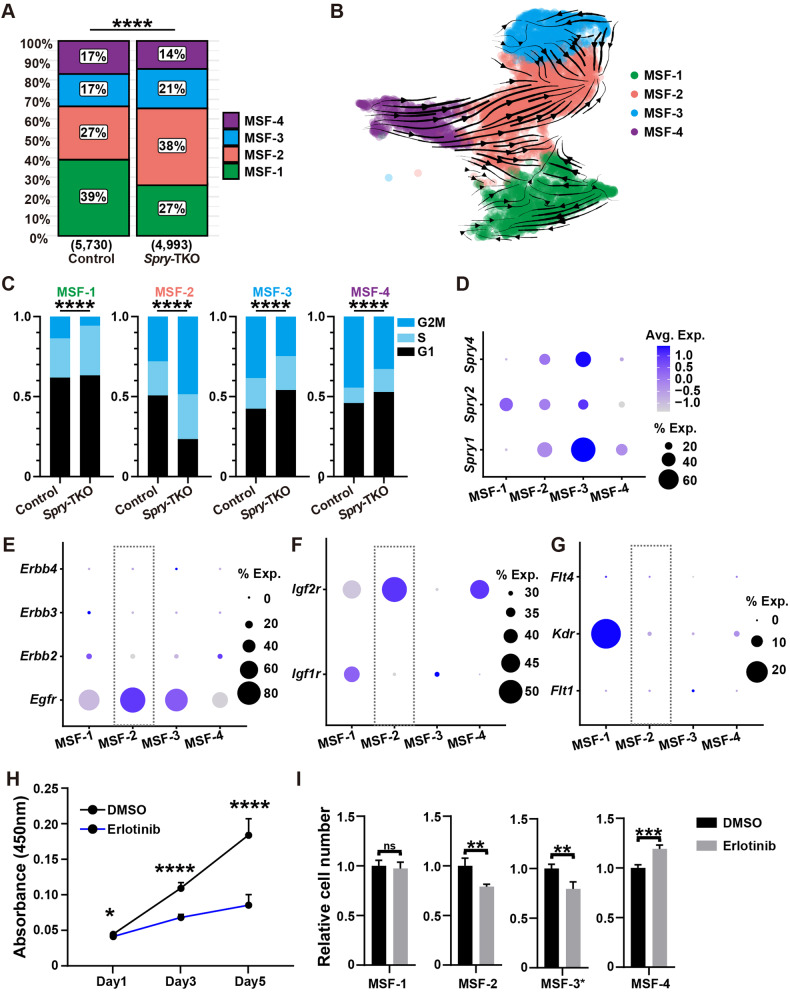
*Spry* genes inhibit expansion of MSF-2 subpopulation. **A** Size distribution of the MSF1-4 subgroups in the control and *Spry-*TKO mammary glands. Numbers in parenthesis indicate sample numbers. **B** Velocity analysis of MSFs in the *Spry-*TKO glands. Note that MSF-2 differentiation from other MSFs were largely the same as in the control gland (Fig. 4H). **C** Cell cycle analysis of cells in the MSF-1 to MSF-4 subgroups from the control and *Spry-*TKO mammary glands. P < 0.001****. Chi-squared test was used for statistical analysis. **D** Expression *Spry1*, *Spry2*, and *Spry4* in MSF1-4 subgroups. **E**–**G** Expression of members of the *Erbb* (**E**), *Igfr* (**F**), and *Vegfr* (**G**) families. Rectangles with dashed lines emphasize RTKs that are expressed by MSF-2 cells. Note that the area of each circle correlates with the percentage of fibroblasts that express the target gene of interest, while changes of color intensity indicate the levels of increased or decreased mRNA expression as in (**D**). **H**, **I** Cell proliferation of total fibroblasts (**H**) or fibroblast subtypes (**I**) treated with medium containing the placebo control DMSO or the EGFR inhibitor Erlotinib. Data were from three independent experiments and were presented as mean ± SD. ns, not significant; *P < 0.05; **P < 0.01; ***P < 0.001, ****P < 0.0001.

Using the marker gene panels, we determined the relative size distributions of the MSFs. We found that MSF-1 was the largest subgroup, comprising 39% of the total fibroblast population; MSF-2 was the second largest at 27% of the total population, whereas MSF-3 and MSF-4 were the third largest, both at 17% of the total population (Fig. 5A). The size of each of the four fibroblast populations was altered in *Spry*-TKO glands, especially MSF-1, which shrank to 27%, whereas MSF-2 increased to 38% in the mutant glands (Fig. 5A). The data are consistent with the observation that increased *Fgf10* production results from a size increase of the *Fgf10-*expressing MSF-2 subpopulation, whereas decreased *Fgf2* and *Fgf7* production results from a size decrease of the *Fgf2-*expression MSF-1 and *Fgf7-*expressing MSF-4 subpopulations. Together, they suggest that *Spry* genes function by regulating the sizes of fibroblast subpopulations in the developing mouse mammary gland stroma.

It is possible that *Spry* loss promotes differentiation into MSF-2 cells. However, analysis of *Spry-*TKO fibroblasts using the velocity program suggested that MSF-2 differentiation from other MSFs was largely normal in the mutant glands (Fig. 5B). Alternatively, *Spry* loss may lead to increased MSF-2 expansion. To test this possibility, we examined the cell cycle states of the MSFs. Consistent with its size increase, we found the percentage of cells in the G2M phase increased from ~28% in control MSF-2 fibroblasts to ~49% in *Spry-*TKO MSF-2 fibroblasts (Fig. 5C). To determine whether *Spry-*TKO cells in the MSF-2 subgroup are more proliferative than control, we isolated them together with control cells from 7-week mammary glands and subjected them to the CCK-8 assay. We observed a significant increase of cell proliferation of *Spry-*TKO cells when compared with control cells of the MSF-2 subgroup (Supplementary Fig. 5A). By contrast, there was little cell death observed in cultured control and mutant MSF subgroups of fibroblasts (Supplementary Fig. 5B–J’).

By contrast, the percentages of cells in the G2M phase dropped in both MSF-1 and MSF-4 subgroups because of *Spry* loss, which are congruent with their size reduction of the two MSFs in the *Spry-*TKO glands (Fig. 5C). Interestingly, the percentage of cells in the G2M phase reduced in the MSF-3 subgroup as a result of *Spry* loss, although its size increased by 4% in the mutant gland. Together, we concluded that *Spry1*, *Spry2*, and *Spry4* inhibit MSF-2 subgroup size during mammary epithelial development, and, in their absence, MSF-2 subgroup expands to a larger than normal size.

Consistent with this notion, we found that *Spry1*, *Spry2*, and *Spry4* mRNA expression was high in the MSF-2 subgroup, as well as the MSF-3 group (Fig. 5D). To examine which RTK is likely to regulate MSF-2 expansion, we mined our scRNA-seq dataset and examined the mRNA expression of each of the main RTKs highlighted by our mass spectrometry data (Supplementary Fig. 2). Of the ERBB family members, we found that *Egfr* is expressed by all the MSFs, including highly in the MSF-2 subgroup, whereas all the other three members are not (Fig. 5E). Likewise, *Igf2r* is highly expressed in the MSF-2 subgroup, while *Igf1r* is not (Fig. 5F). None of the members of the VEGFR family are highly expressed in the MSF-2 subgroup (Fig. 5G). The results thus suggest that both EGFR and IGF2R are candidate RTKs that regulate MSF-2 expansion.

Next, we chose EGFR as the candidate to test whether it plays a role in regulating MSF-2 expansion. To this end, we harvested mammary fibroblasts and cultured them in growth medium with control (DMSO) or the EGFR inhibitor Erlotinib. We found that Erlotinib potently inhibited fibroblast growth when compared with DMSO control (Fig. 5H). Moreover, when added to medium culturing individual MSF subgroups, Erlotinib also inhibited proliferation of both the MSF-2 and MSF-3 subgroups, although an increased cell proliferation was observed in the MSF-4 subgroup (Fig. 5I).

Together, the above data show that *Spry1*, *Spry2*, and *Spry4* inhibit MSF-2 expansion by modulating RTK signaling and, in their absence, MSF-2 expansion increases, leading to excessive FGF10 production and accelerated epithelial branching.

### MSF-2 subgroup consists of activated fibroblasts

To determine whether MSF subtypes have distinct functions, we performed GO and KEGG analysis using the scRNA-seq data we acquired above. In both cases, we found that the MSF subtypes share some signaling pathways, but also showed subtype-specific signaling activities, which together formed a subtype-specific combination of signaling pathways (Supplementary Fig. 6A, B). Such intrinsic differences among the MSF subtypes could mean differences in readouts by each MSF in the presence of similar signaling stimulations.

To test this possibility, we set out to develop a FACS strategy to enrich each MSF subpopulation. We successfully used CD26 (DPP4) for enriching MSF-1 cells, CD36 for MSF-2 cells, and CD142 (F3) for MSF-4 cells. However, we did not find an antibody that could satisfactorily enrich MSF-3 subtype cells. As such, we refer to fibroblasts that were not MSF-1, MSF-2, or MSF-4 as MSF-3*. Briefly, after removal of lineage-positive cells, namely endothelial cells, blood cells, and immune cells, we sorted for fibroblasts using a PDGFR antibody (Supplementary Fig. 7A, B; see methods for details). CD26 and CD142 antibodies were then used to enrich MSF-1 and MSF-4, respectively, while CD36 antibody was used to enrich for MSF-2 cells from the CD26^-^CD142^-^ cells. Using Western blotting analysis, we first confirmed that CD26, CD36, and CD142 were enriched in the MSF-1, -2, and -4 subgroups, respectively (Supplementary Fig. 7C).

**Fig. 6:**
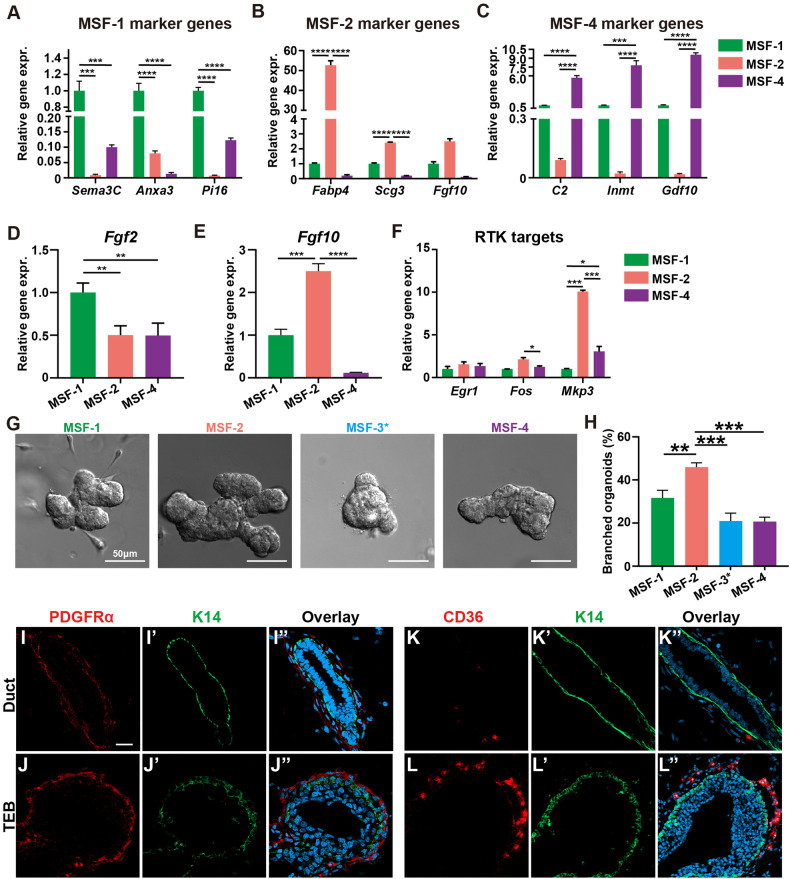
MSF-2 subgroup consists of activated fibroblasts. **A**–**C** mRNA expression as detected by qPCR of several genes in the marker panel of the MSF-1 (**A**), MSF-2 (**B**), and MSF-4 (**C**) to validate whether subgroup cells have been specifically enriched by FACS. Expr, expression. **D**–**F** mRNA expression as detected by qPCR of *Fgf2* (**D**) and *Fgf10* (**E**) or several RTK signaling target genes (**F**) in the MSF-1, MSF-2, and MSF-4 subgroups to validate whether subgroup cells have been specifically enriched by FACS. **G**, **H** Wildtype organoids co-cultured with fibroblasts from the MSF-1 to MSF-4 subgroups (**H**) with their branching ratios quantified (**I**). *N* = 3. Scale bars: 50 μm. Data were from three independent experiments. Plots show mean ± SD (*n* $\ge$3); ns, not significant; *P < 0.05; **P < 0.01; ***P < 0.001; ****P < 0.0001. Scale bars: 50 μm. **I**–**L** Confocal images of immunofluorescence of PDGFR (**I**, **J**), K14 (**I’**, **K’**, **J’**, **L’**), CD36 (**K**, **L**), and overlay, marking pan-fibroblasts and MSF-2 fibroblasts, respectively, around mammary ducts (**I**, **K**) or TEBs (**J**, **L**) using frozen sections of mammary glands at the 7-week stage. Note blue nuclei in the overlayed images. Scale bars: 20 μm.

To determine whether the sorted cells were indeed the expected MSF subtypes, we performed qPCR reactions using a panel of marker genes specific for each MSF. We found that the MSF-1 panel of marker genes, including *Sema3c*, *Anxa3*, and *Pi16*, were specifically enriched in MSF-1, but not MSF-2 or MSF-4 cells (Fig. 6A); whereas the use of MSF-2 and MSF-4 panels of marker genes were able to specifically enrich cells of the corresponding subtypes (Fig. 6B, C). We also examined mRNA expression of both *Fgf2* and *Fgf10* and confirmed that they were specifically enriched in MSF-1 and MSF-2 subtypes, respectively (Fig. 6D, E). Thus, our FACS strategy was able to specifically enrich three out of four subtypes of mammary stromal fibroblasts. Finally, we found that the mRNA expression of ERK signaling target genes, *Egr1*, *Fos*, and *Mkp3*, showed differing levels in the MSF subtypes such that ERK signaling was higher in MSF-2 subtype than in MSF-1 and MSF-4 subtypes (Fig. 6F).

Such molecular differences imply that distinct functions exist among the MSF subtypes. To test this possibility, we used the cells from each MSF subtype and performed co-culture branching experiments together with wild-type organoid epithelium as mentioned above. We found that the MSF-2 was the most potent subtype to stimulate organoid branching, while MSF-1 came second, and with MSF-4 and MSF-3* had the least potency to stimulate organoid branching (Fig. 6G, H).

The data so far show MSF-2 is a special subgroup, being derived from the other MSFs and most potent in its ability to promote epithelial branching. We therefore next investigated the spatial localization of MSF-2 fibroblasts. Using immunofluorescence microscopy, we first confirmed that cells expressing PDGFR, a pan-fibroblast marker, were distributed throughout the mammary gland, particularly around the periductal epithelium of both the ducts and terminal end buds (TEBs) (Fig. 6I, J”). Notably, MSF-2 fibroblasts were predominantly located adjacent to TEBs and were absent from periductal stroma (Fig. 6K, L”).

Given that active epithelial invasion and branch-point formation occur at TEBs, and that there appears to be a unique function of MSF-2 cells, we propose that these constitute a specialized “activated” fibroblast subpopulation that actively interacts with mammary epithelial cells to contribute to essential morphogenetic processes. The data so far suggest that there are more activated MSF-2 subgroup cells in *Spry-*TKO glands. Using immunofluorescence to detect MSF-2 cells, we confirmed that activated fibroblasts are not present around either control or *Spry-*TKO epithelium, and that there are no ectopic activated peri-ductal fibroblasts in the mutant gland (Supplementary Fig. 8A, B”’).

### *Spry* downregulation and activated fibroblast expansion in breast cancer

**Fig. 7:**
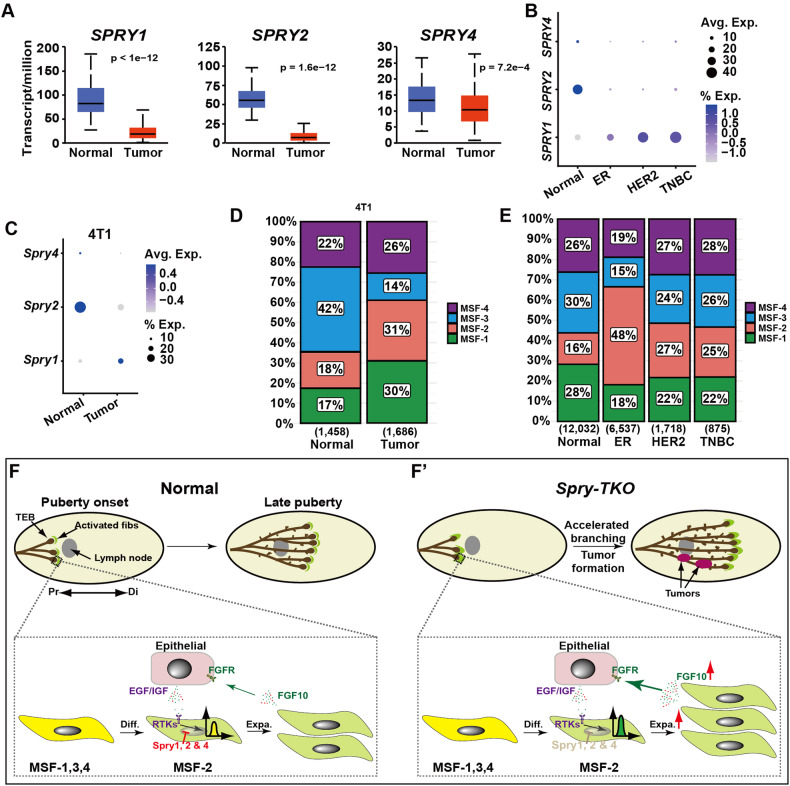
*Spry* downregulation and activated fibroblast expansion in breast cancer. **A** *Spry1*, *Spry2*, and *Spry4* mRNA expression in human breast cancer subtypes using the TCGA database. (**B**, **C**) *Spry1*, *Spry2*, and *Spry4* mRNA expression in stromal fibroblasts of human breast cancer samples (**B**) or a mouse model of breast cancer using the 4T1 cells (**C**). **D**, **E** Size distribution of the MSF1-4 subgroups in the control and 4T1 cancer fibroblasts of the mouse (**D**) or human breast cancer (**E**). Numbers in parenthesis indicate sample numbers. **F**, **F’** Schematic diagram of the process of differentiation and expansion of activated fibroblasts in mammary gland development and breast cancer. **F** Activated fibroblasts of the MSF-2 subgroup are derived from progenitors in the MSF-1, MSF-3, and MSF-4 subgroups, presumably due to interactions between the stromal microenvironment and the epithelium actively undergoing invasion and branch-formation. Epithelial-derived ligands of the EGF and IGF families further promotes MSF-2 subgroup expansion via RTK signaling, which is negatively regulated by the *Spry* genes. Activated fibroblasts in the MSF-2 subgroup are responsible for the production of branching factors, e.g., FGF10, to promote epithelial development in the mammary gland. **F’** Upon downregulation of *Spry* functions, e.g., when the *Spry* genes are knocked out in the mutant mammary glands or their functions are otherwise silenced in breast cancer, multiple pathways of RTK signaling, especially EGFR and IGFR signaling are increased. This leads to an over-expansion of the activated fibroblast population and, consequently, over-production of branching factors such as FGF10. As a result, epithelial development is accelerated, as manifested in precocious branching morphogenesis in *Spry-*null glands or breast cancer development in mice and humans: Diff. Differentiation, Expa. Expansion, TEB terminal end bud.

Due to their role in RTK signaling inhibition, *Spry* genes are considered as candidate tumor suppressors. Given our findings detailed above, we set out to test this hypothesis, especially with regards to whether changes of MSF subpopulations due to the loss of *Spry* genes may promote breast cancer progression. Therefore, we first examined *Spry1*, *Spry2*, and *Spry4* mRNA expression in The Cancer Genome Atlas (TCGA) database. We found that all three *Spry* genes are downregulated in breast cancer (Fig. 7A). Next, we searched the database of a recent scRNA-seq analysis on the tumor microenvironment of human breast cancer [35]. Focusing on the mRNA expression in the stromal fibroblasts, we found that *Spry2* and *Spry4* are both greatly downregulated, but curiously, *Spry1* is upregulated in the cancer fibroblasts (Fig. 7B).

scRNA-seq has recently been performed on the stromal microenvironment of breast cancer using a 4T1 mouse model [36]. Therefore, we mined the database and confirmed that expression of both *Spry2* and *Spry4* is downregulated, while *Spry1* is upregulated in the cancer fibroblasts of the 4T1 model, similar to that in the human cancer (Fig. 7C). Furthermore, we found that MSF-1 and MSF-2, the two largest subpopulations out of the four, showed a decrease and an increase, respectively, in population size, which was very similar to *Spry-*TKO mammary glands (Fig. 7D). Interestingly, we found that the MSF-2 subgroup size is larger than normal (31% vs. 18%) in the 4T1 scRNA-seq dataset, similar to the data in which *Spry* genes were lost. Likewise, the MSF-2 subgroup size is also enlarged in all the three subtypes, including the ER, HER2, and TNBC subtypes of human breast cancer (Fig. 7E).

Together, these data show that *Spry* genes are downregulated, while the MSF-2 subpopulation is increased in both mouse models and human breast cancer, suggesting that they play an important role in cancer progression, as well as in development.

## Discussion

The microenvironment of vertebrate organs is critical for their development, physiology, and pathology, with stromal fibroblasts being a significant component. Recent advances in single-cell techniques have revealed that fibroblasts are heterogeneous, leading to questions about their origin, differentiation, and function. Here, we demonstrate that the loss of *Spry1*, *Spry2*, and *Spry4* in mammary stromal fibroblasts increases signaling in multiple RTK pathways and leads to enhanced ECM remodeling, which accelerates epithelial branching. We found that the increased production of FGF10 due to *Spry* loss is due to an over-expansion of a functionally distinct subgroup of fibroblasts with potent branching-promoting ability, which are “activated” and located next to actively branching and invading epithelium. Importantly, *Spry* genes are downregulated, and activated fibroblasts are expanded in all three breast cancer subtypes. These findings highlight the essential role of *Spry* genes in the regulation of a functional subtype of mammary fibroblasts and their critical role in epithelial morphogenesis and cancer development.

### Stromal *Spry* genes regulate epithelial branching by controlling subgroup size of activated fibroblasts

Our results show that activated fibroblasts of the MSF-2 subgroup are derived from progenitors in the MSF-1, MSF-3, and MSF-4 subgroups, presumably due to interactions between the stromal microenvironment and the epithelium actively undergoing invasion and branch-formation. Epithelial-derived ligands of the EGF and IGF families further promote MSF-2 subgroup expansion via RTK signaling, which is negatively regulated by the *Spry* genes. Activated fibroblasts in the MSF-2 subgroup are responsible for the production of branching factors, e.g., FGF10, to promote epithelial development in the mammary gland (Fig. 7F). Upon downregulation of *Spry* functions, e.g., when the *Spry* genes are knocked out in the mutant mammary glands or their functions are otherwise silenced in breast cancer, multiple pathways of RTK signaling, especially downstream of EGFR and IGFR, are increased. This leads to an expansion of the activated fibroblast population and, consequently, over-production of branching factors such as FGF10, which we reported as the most important stromal FGF ligand regulating epithelial morphogenesis in the mammary gland [25, 27, 28]. As a result, epithelial development is accelerated, as manifested in precocious branching morphogenesis in *Spry-*null glands or breast cancer development in mice and humans (Fig. 7F’).

We do not exclude the possibility that the differentiation process of activated fibroblasts of the MSF-2 subgroup may also be regulated by RTK signaling or *Spry* functions. This is because the *Fsp1*-Cre transgene may be activated after MSF-2 subgroup differentiation is completed. Previous reports suggest that fibroblast differentiation in the human dermal fibroblasts is regulated by FGF signaling [37]. As FGF signaling is regulated *by Spry* genes in various developmental contexts, including mammary gland epithelial morphogenesis, it is possible that *Spry* gene products may regulate MSF-2 differentiation in the mammary stroma. A definitive answer to this question will require removal of *Spry* genes prior to the differentiation onset of MSF-2.

Moreover, our discovery that FGF10 is over-produced as a result from an increase of the number FGF10-producing MSF-2 cells, rather than its transcriptional upregulation, is consistent with accelerated epithelial branching. However, FGF10 is likely only one of the various branch-promoting factors in the stroma. Indeed, our data show that paracrine factors, to which FGF10 belongs, account for only around 50% of the branch-promoting effects from stromal fibroblasts. Future studies will address what the non-paracrine factors are that also facilitate epithelial branching from the stromal fibroblasts.

Our findings are consistent with the notion that *Spry* genes act as negative regulators of RTK signaling. Interestingly, other stromal factors that negatively regulate epithelial branching including *Cbl*, which is an adaptor protein inhibiting the RTK signaling pathway, and *Tgf1b* are not affected by *Sprouty* loss, suggesting that they may function in parallel or independently in the stroma to regulate epithelial branching [29, 30].

It is noteworthy that although in vitro experiments indicated that *Spry* genes can inhibit various RTK signaling pathways, early reports from genetic studies showed that *Spry* primarily regulates FGF signaling, including in the mammary epithelium as we reported [25, 38]. However, subsequent studies show that *Spry* also regulates other RTKs, including EGFR in mammary gland stroma and GDNF/Ret in the kidney [12, 24]. In the current study, we expand the range of RTKs that can be controlled by *Spry* genes and thus support the previous in vitro data on *Spry* regulation and emphasize that *Spry* genes may regulate one or more RTK signaling pathways, depending on spatiotemporal controls in different developmental contexts.

### Origin and function of fibroblast subtypes

Our scRNA-seq data indicate that there are four subtypes of fibroblasts in the mammary gland stroma that show distinct patterns of gene expression, including *Spry* genes and components of the RTK signaling pathways. Functional analysis further show that the MSF-2 subgroup is unique in that not only its fibroblasts are derived from the three other developmentally independent subgroups, but they have the most potent branching-stimulating ability and are specifically localized to the epithelial invasion front.

Our data are consistent with previous reports from lineage tracing studies showing that mammary gland fibroblasts are derived from several independent sources [39]. They also support the notion that these subtypes may be functionally distinct. However, it is noteworthy that such suggestions have been mainly based on the assumption that the differences in gene expression patterns, as observed in different subtypes, reflect their functional distinctions [40]. Although our data, based on the in vitro branching assay, provide the first experimental evidence that fibroblast subtypes are functionally distinct, this conclusion needs to be rigorously tested in future in vivo studies.

### Targeting activated fibroblasts in cancer treatment

We found that the activated fibroblast subpopulation in the mammary stromal fibroblasts is increased due to *Spry* loss, which leads to upregulated EGFR and IGF2R signaling essential for the expansion of MSF-2. Interestingly, the *Spry* genes are not only downregulated in breast cancer cells, as indicated by the TCGA data, but in the CAFs based on our single cell transcriptomics analysis. Our data are thus consistent with the notion that RTK signaling often promotes cancer development and that *Spry* genes are tumor suppressors of breast cancer.

Interestingly, the MSF-2 subpopulation is increased in all three subtypes of breast cancer, like its increase due to the removal of *Spry* genes. It is thus tempting to speculate that *Spry* genes normally inhibit cancer progression in mammary fibroblasts by reducing the subgroup size of the activated fibroblasts in the MSF-2 population. In the absence of *Spry* genes, as a result of genetic removal or other silencing mechanisms during tumorigenesis, the MSF-2 subpopulation is expanded and becomes larger than normal. This leads to an overproduction of bioactive factors including FGF10, which in turns causes excessive epithelial development and possibly cancer development.

It remains unclear whether Sprouty genes function at one or more stages of breast cancer development, including ductal carcinoma in situ (DCIS) and metastasis. Considering that Sprouty loss in the stroma leads to epithelial over-expansion, and its loss is ubiquitously found in breast cancer subtypes of different invasiveness, with TNBC subtype being the most aggressive, it is tempting to conclude that Sprouty genes function at multiples stages of normal development of the mammary gland and breast cancer progression. However, future studies are need to future examine these possibilities.

In conclusion, the study highlights the critical role of the stromal microenvironment and fibroblast-epithelial interactions in mammary gland formation and cancer development. The findings provide new insights into the regulation of a functionally distinct subgroup of fibroblasts by *Spry* genes and their essential role in epithelial morphogenesis and cancer development. Furthermore, the study emphasizes the importance of understanding the function of distinct fibroblast subtypes for the development of effective cancer therapies targeting the tumor microenvironment.

## Materials and methods

### Mouse strain

Mice carrying the *Fsp-Cre* allele [26] and the MMTV*-*PyMT (FVB) allele [41] were purchased from the Jackson Laboratory. The genotype identification of mice was carried out according to the previous method [12], and the primer sequences used were as follows: Fsp-cre (Forward primer: TCAGCAACACATATCCAGTTCTC; Reverse primer: GGCAAACGGACAGAAGCA), Spry1 (Forward primer: CTCAATAGGAGTGGACTGTGAAACTGC; Reverse primer: GGGAAAACCGTGTTCTAAGGAGTAGC), Spry2 (Forward primer: AATAGGGATTGTTGCTCCG; Reverse primer: GCATGGGCTATTCACAAAC), and Spry4 (Forward primer: CAGGACTTGGGAGTGCTTCCTTAG; Reverse primer: CCTCCTAGTACCTTTTTGGGGAGAG). No statistical method was used to predetermine the sample size for mice experiment. The sample size of each experiment is shown in the legend. No data were excluded from the analysis. No blinding method was used for mice experiment.

### Mammary gland staining and histology

The fourth pair of mammary glands were harvested and mounted on glass slides for Carmine staining. After overnight fixation in Carnoy’s fixative at 4 °C, they were hydrated and stained in Carmine alum for 4 h. The stained mammary glands were then dehydrated and cleared in Histoclear before being photographed using a Leica M205 stereoscope fitted with a Leica DFC345 FX camera. The resulting photographs were processed and analyzed using GIMP and Image J (NIH). Ductal length was assessed as the distance between the tip of the most distally reaching branch and the center of the lymph node.

### Preparation of primary mammary organoids and fibroblasts

To prepare primary mammary organoids and fibroblasts, mouse mammary glands were finely chopped, and the mince was digested in collagenase buffer [0.2% collagenase (Sigma) and 0.2% trypsin (Life Technologies) in DMEM/F12 supplemented with 5% FBS, 50 µg/ml gentamicin, 5 µg/ml insulin (all from Sigma)] for 30 min at 37 °C. The resulting suspension was centrifuged, the pellet was washed with DMEM/F12, and then treated with DNase I (2 U/μl) for 5 minutes. After another wash with DMEM/F12, the pellet was resuspended in DMEM/F12, and the sample was subjected to a short-pulse centrifugation at 500 g (differential centrifugation). The supernatant was collected, and the pellet was resuspended in DMEM/F12 for another round of differential centrifugation. After five rounds of differential centrifugation, the final pellet containing mammary organoids was plated in Matrigel (Corning) and overlaid with basal organoid medium (DMEM/F12, 10 μg/ml insulin, 5.5 μg/ml transferrin, 6.7 ng/ml selenium, 100 U/ml penicillin, 100 μg/ml streptomycin).

The supernatant fractions were pooled, centrifuged, and the resulting pellet was resuspended in DMEM (Invitrogen) with 10% FBS (Sigma), 10 μg/ml insulin, 5.5 μg/ml transferrin, 6.7 ng/ml selenium, 100 U/ml penicillin, and 100 U/ml streptomycin (fibroblast medium), and then seeded onto a cell culture dish. After 30 min, when the fibroblasts had adhered to the dish but other cellular types remained in suspension, the medium was aspirated, and the cell culture dish was washed twice with PBS before adding fresh medium. The fibroblast cultures were allowed to grow until they reached approximately 80% confluence, and then they were sub-cultured. Only early passage fibroblasts (up to passage number 5) were used in experiments.

Transfection: Fibroblasts were resuspended in fibroblast medium and infected overnight with Adenovirus-Cre-GFP (green fluorescent protein) [42] at a multiplicity of infection of ~25 particles per cell. The next day, the fibroblasts were washed twice with PBS and supplied with fresh fibroblast medium. After counting the fibroblasts, the adenovirus was added, and the cells were plated in a 96-well low adsorption plate (CORNING#3474) for 2 h. The adenovirus was custom-made by Genkai Genetics.

### Mammary epithelial organoids co-cultured with fibroblasts

Fibroblasts and epithelial organoids were counted separately, and the ratio of the two was maintained at approximately 1:100. They were then resuspended in Growth Factor Reduced-Matrigel and plated in 24-well plates (Thermofisher #174930). The medium was changed on the fourth day, and photographs were taken.

### RNA isolation and qPCR

RNA was isolated from the cells or tissues using either the RNAqueous® kit from Life Technologies or the RNeasy Mini kit from Qiagen. The isolated RNA was then used to prepare cDNA using TaqMan® Reverse Transcription Reagents from Life Technologies. Real-time qPCR was performed using SYBR® Green PCR Master Mix from Life Technologies and 0.45 µM of gene-specific primers in a final volume of 11 µl. The qPCR reactions were performed using the ABI 7900HT or QuantStudio™ 12 K Flex Real-Time PCR System from Life Technologies. The oligonucleotide sequences used for the gene-specific primers are described in Table S1. The gene expression data were then normalized to housekeeping genes, either β-actin (*Actb*) or eukaryotic translation elongation factor 1 gamma (*Eef1g*).

### In vitro epithelial branching assay

Freshly extracted mammary organoids were plated in Matrigel and cultured in basal medium containing growth factors of interest (2.5 nM FGF2 or FGF7, Sigma) for 10 days. Every 3 days, the medium was changed for fresh medium. For organoid-fibroblast co-cultures, CD-1 organoids were mixed with fibroblasts at a ratio of 100 fibroblasts per organoid, plated in Matrigel, and cultured in the basal medium (supplemented with 5 nM FGF2 when necessary) for 5 days.

### Western blotting

The fibroblasts were starved in serum-free medium for 24 h, and then stimulated with various RTK ligands for specific time periods. The medium was then discarded, the cells were washed twice with cold PBS, and protein lysate was added. The lysate contained RIPA buffer (Beyotime # P0013J) and inhibitors (Bimake #B15001, #B14001), and was kept at 4 °C for 25 min. After centrifugation, the supernatant was collected, and protein quantification was performed using a kit (Thermoscientific #23225). Gel running was performed on a 10% precast gel (Meilun e# MA0295) and transferred to film. The membrane was blocked with 5% BSA (sigma # WXBC3116V) at room temperature for 2 h. The primary antibody was incubated overnight at 4 °C, and the secondary antibody was incubated at room temperature for 2 h. Chromogenic solution (Epizyme# SQ101L) was added and the film was developed using the Amersham Imager 680. Primary antibodies used were ERK1/2 (CST#9102, 1:1000), P-ERK1/2 (CST#9101, 1:1000), GAPDH (ABclonal#AC033, 1:20000). Secondary antibodies used were anti-Rabbit-HRP (CST#7074 S, 1:2000), anti-Mouse-HRP (CST#7076 S, 1:2000). All antibodies were diluted with 5% BSA.

### Collagen contraction assays

To prepare the collagen gel, collagen type I (3.9 mg/ml; Corning) was combined with collagen reconstitution buffer (5x MEM, 20 µg/ml NaHCO3, 0.1 M HEPES) in a ratio of 12.5:2.5. To this mixture, 1 volume of 0.22 M NaOH, 3.1 volumes of FBS, and 3.1 volumes of fibroblasts in DMEM (2x106 fibroblasts/ml) were added, resulting in a final collagen concentration of 2.2 mg/ml. The gel-fibroblast mixture was then plated in equal volumes in a 24-well plate and allowed to set at 37 °C for 1 h before adding fibroblast medium (with or without inhibitors). Samples were fixed in 4% PFA after 4 days of culture, and images were captured using a SteREO LumarV12 stereoscope. The images were analyzed using ImageJ software.

### FACS

After the extraction of fibroblasts, they were incubated with antibodies and left to sit at 4 °C for 30 min. Subsequently, the antibodies were washed off, and the cells were resuspended and sorted using a BD Aria III flow cytometer. The antibodies used for staining were as follows: anti-CD31 (BioLegend #102417), anti-ter119 (BioLegend #116221), anti-CD45 (BioLegend #100432), anti-CD140a (BioLegend #135907), anti-CD142 (Sino Biological #50413-RP01), anti-CD26 (BioLegend #137804), and anti-CD36 (ThermoFisher #56-0362-82). FITC coupling was required for the anti-CD26 antibody, and both antibodies were used at a 1:100 dilution.

### Mass spectrometry sample preparation and analysis

The *Spry1*, *2*, *4*^fl/fl^ and *Spry1*, *2*, *4* KO mammary fibroblasts were washed with PBS and scraped into a centrifuge tube using a cell scraper. The cells were then disrupted on ice using ultrasonication (10 s on, 10 s off, for 3 cycles). The resulting peptides were subjected to overnight enzymatic digestion, and then stored at −80 °C until further analysis. The peptides were subsequently analyzed using LC-MS.

### Single cell RNA sequencing and bioinformatics analysis

Two mice each for the control and Spry null mice at the 7-week stage were used to prepare mammary epithelial cells and stromal fibroblasts. Dissociated cells of the same genotype were combined and used for scRNA-seq. After quality screening and validation, 5730 cells from control glands and 4993 cells from mutant glands were used for the analysis. We used the Cell Ranger analysis pipeline (version 6) to process 10X Chromium single-cell data and R package (version 4.1.1) for downstream analysis and evaluation of scRNA-seq data collected from the experiment [43]. The selection criteria used in this study involved the removal of cells that had a detected gene count of less than 200 or greater than 5900, mitochondrial gene expression greater than 7, and ribosomal gene expression greater than 49. For functional enrichment analysis, we used the ClusterProfiler R package to perform GO and GSEA analysis [44]. We used the CellChat R package for cell communication analysis, with a minimum expression of at least ten cells used as the criterion for selecting receptors and ligands [45]. RNA velocity analysis was performed using scVelo [34]. Pseudotime analysis was performed using monocle [46].

We downloaded human breast cancer data from GSE161529 and selected patient samples containing fibroblasts, obtaining a total of 57 patient data. We selected the top 100 differentially expressed genes from four subtypes in our data to construct gene sets and used the GSVA package for ssGSEA analysis to obtain scores for the four gene sets in each cell. The cell subtype represented by the gene set with the highest score was considered the cell type of that cell. For the analysis of 4T1 breast tumors, we obtained relevant data from (https://datadryad.org, 10.6071/M3238R), and identified fibroblast subtypes in 4T1 using the same method as for human breast cancer as described above.

### Statistical analyses

No statistical methods were used to predetermine the sample size. Statistical analyses were carried out using Prism software (GraphPad Software). Graphs showing mean values and standard deviations (SD) were generated from multiple repetitions of biological experiments. *P*-values were obtained from t-tests with unpaired samples or from ANOVA. Symbols indicating the level of significance are as follows: *P < 0.05; **P < 0.01; ***P < 0.001; ****P < 0.0001.

### Supplementary information

Full and uncropped Western Blots

Supplementary Table

Supplementary Figures and Legends

## Data Availability

All data supporting the conclusions of the paper are available in the article and corresponding figures. scRNA-seq data and human breast cancer data were downloaded from public databases as indicated in the methods section.
