# Supplementary figures and images for: Sprouty genes regulate activated fibroblasts in mammary epithelial development and breast cancer
Source: Cell Death Dis. 2024 Apr 10;15(4):256. doi: 10.1038/s41419-024-06637-2 (PMC11006910; doi:10.1038/s41419-024-06637-2)

**Fig. S2C**

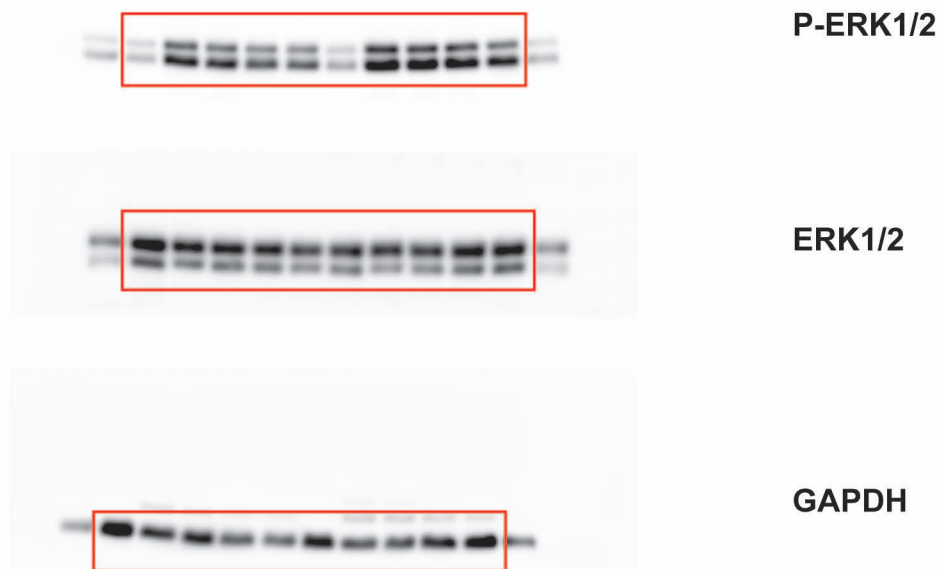

**Fig. S2E**

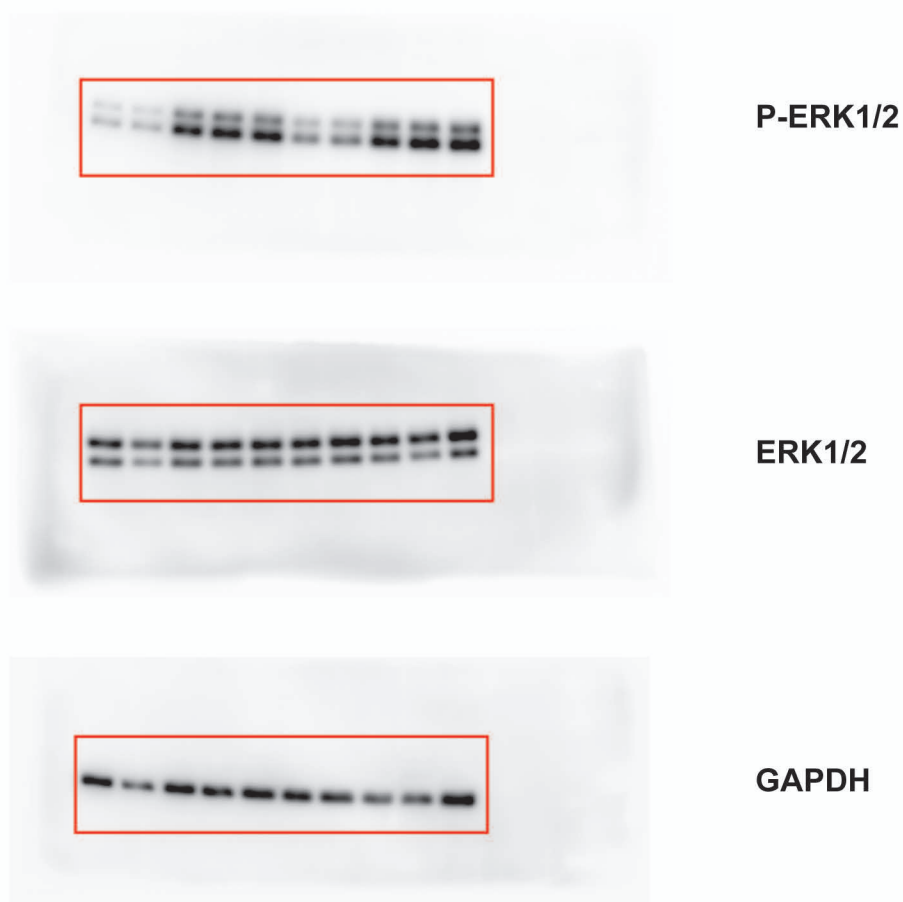

**Fig. S2G**

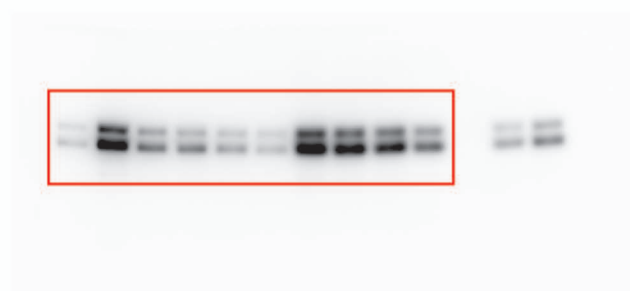

**P-ERK1/2**

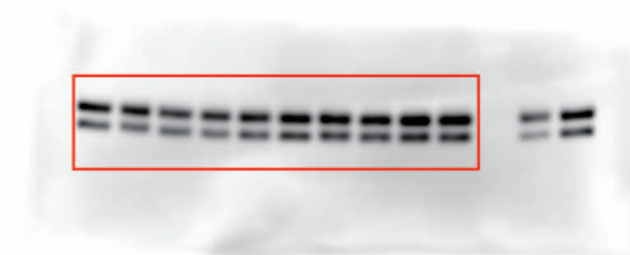

**ERK1/2**

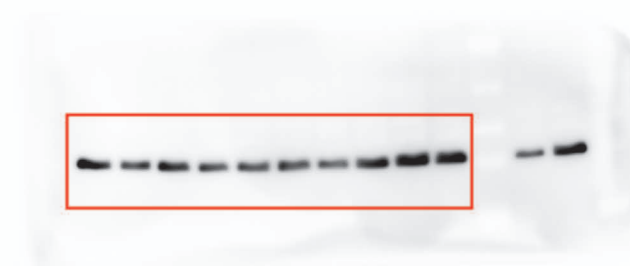

**GAPDH**

Supplement: Supplementary file 1 — Full and uncropped Western Blots [file 41419_2024_6637_MOESM1_ESM.pdf]
