# Supplementary material for: Sprouty genes regulate activated fibroblasts in mammary epithelial development and breast cancer
Source: Cell Death Dis. 2024 Apr 10;15(4):256. doi: 10.1038/s41419-024-06637-2 (PMC11006910; doi:10.1038/s41419-024-06637-2)
Supplement: Supplementary file 2 — Supplementary Table [file 41419_2024_6637_MOESM2_ESM.docx]

SUPPLEMENTARY TABLE1. Primers used in qPCR.

| Gene name | Forward sequence (5’ 🡪 3’) | Reverse sequence (5’ 🡪 3’) |
| --- | --- | --- |
| *Actin* | ggctgtattcccctccatcg | ccagttggtaacaatgccatgt |
| *Spry1* | ggtcataggtcagatcgggtc | cttgccacactgttcgcag |
| *Spry2* | tccaagagatgcccttaccca | gcagaccgtggagtctttca |
| *Spry3* | gtaaacaagctctttctagccct | agtggtgctttgggacattga |
| *Spry4* | gcagcgtccctgtgaatcc | tctggtcaatgggtaagatggt |
| *Egr1* | agcgaacaaccctatgagcac | tcgtttggctgggataactcg |
| *Fos* | aagggaacggaataagatggc | caacgcagacttctcatcttcaa |
| *Lox* | tcttctgctgcgtgacaacc | gagaaaccagcttggaaccag |
| *Etv4* | cggaggatgaaaggcggatac | tcttggaagtgactgaggtcc |
| *Etv5* | tcagtctgataacttggtgcttc | ggcttcctatcgtaggcacaa |
| *Mkp3* | tcgggctgctgctcaagaaac | cggtcaaggtcagactcaatgtcc |
| *Fgf2* | cggctctactgcaagaacg | tgcttggagttgtagtttgacg |
| *Fgf7* | tgggcactatatctctagcttgc | gggtgcgacagaacagtct |
| *Fgf10* | tttggtgtcttcgttccctgt | tagctccgcacatgccttc |
| *Igf1* | tcggcctcatagtacccact | acgacatgatgtgtatctttattgc |
| *Mmp2* | caagttccccggcgatgtc | ttctggtcaaggtcacctgtc |
| *Mmp3* | ttgttctttgatgcagtcagc | gatttgcgccaaaagtgc |
| *Mmp13* | gccagaacttcccaaccat | tcagagcccagaattttctcc |
| *Fabp4* | aaggtgaagagcatcataaccct | tcacgcctttcataacacattcc |
| *Scg3* | gaatctgctgagggcaataaca | cagcgtcttccacgttcagtt |
| *Fgf10* | tttggtgtcttcgttccctgt | tagctccgcacatgccttc |
| *Pi16* | ggggccacaacaaagaacg | cacatctggttcggatcgca |
| *Anxa3* | atggcctctatctgggttgga | caagtcctctgatcgctttcc |
| *Sema3c* | atggcattccgggcgattt | ggttttggtttctcgaagctca |
| *Gdf10* | caggacatggtcgctatccac | acaggcttttggtcgatcatttc |
| *Inmt* | gcagagcaggaaatcgtaaagt | ggggtgtagtcagtgacaatgat |
