# Supplementary material for: Sprouty genes regulate activated fibroblasts in mammary epithelial development and breast cancer
Source: Cell Death Dis. 2024 Apr 10;15(4):256. doi: 10.1038/s41419-024-06637-2 (PMC11006910; doi:10.1038/s41419-024-06637-2)

Figure S1. Loss of *Spry1*, *Spry2*, and *Spry4* in the mammary stromal fibroblasts causes accelerated epithelial branching.

(**A**) *Spry1,2,4* mRNA expression as detected by quantitative RT-PCR (qPCR). (**B**) *Spry1,2,4* mRNA expression was measured by qPCR. mRNA was harvested from wild-type and *Spry1,2,4*-KO fibroblasts. At least three mice were used for each stage. (**C**) Quantification of the number of TEBs. Graph data were from three independent experiments and show mean ± SD. ns, not significant; ***, P<0.001.

Figure S2. Multiple RTK signaling pathways in the fibroblasts are sensitized due to the loss of *Spry1*, *Spry2*, and *Spry4*.

(**A**) Schematic diagram depicting the experimental procedures during sample preparation, in vitro culture and treatment methods of protein phosphorylation mass spectrometry. Mammary organoids and stromal fibroblasts were prepared from wild type (WT) and *Spry1*$^{fl/fl}$; *Spry2*$^{fl/fl}$; *Spry4*$^{fl/fl}$ mice. Fibroblasts with and without *Spry1*, *Spry2*, and *Spry4* function were acquired by infection with Ad-Cre-GFP and FACS-mediated selection of GFP-positive infected cells. Control and *Spry1*,*2*,*4*-KO fibroblasts were then subjected to procedures for mass spectrometry (see methods for details).

(**B**) Changes in RTK pathways, as reflected by up- or down-regulation of the phosphorylation/activation status of the key signaling components in *Spry1*,*2*,*4*-KO fibroblasts when compared with control fibroblasts.

(**C-H**) Western blot analysis of the phosphorylation status of ERK1/2 in Control and *Spry1,2,4*-KO fibroblasts in response to FBS (**C-D**), VEGF (**E-F**) or EGF (**G-H**) stimulation. Fibroblasts were serum-starved, treated with FBS, 5µg/ml VEGF or 10ng/ml EGF for the indicated durations, lysed and analyzed on a single blot, p-ERK1/2 (Thr202/Tyr204), ERK, and GAPDH signals were detected. (**D, F, H**) Quantitative comparison of ERK1/2 phosphorylation, normalized to total ERK1/2. Data are mean ± SD (n = 3). Statistical analysis was performed using unpaired student t test; ns, not significant; *, P<0.05; **, P<0.01; ***, P<0.001.

Figure S3. Bioinformatic analysis of *Cbl* and *Tgfb1* mRNA expression.

(**A**, **B**) Analysis of *Cbl* (**A**) and *Tgfb1* (**B**) mRNA expression in control and mutant subgroups of mammary fibroblasts. No difference was detected.

Figure S4. MSF-2 subgroup is derived from three independent fibroblast lineages.

(**A**) Heat map showing relative log-expression of the top 10 marker genes, with each gene indicated, for each cell cluster identified in Fig. 4A.

(**B**) Pseudotime analysis of mammary stromal fibroblast differentiation trajectory.

Figure S5. Cell proliferation and TUNEL analysis of mammary stromal fibroblasts.

(**A-H'**) Cell proliferation of control and *Spry*-TKO fibroblasts of the MSF-2 subgroup from 7-week mammary glands as analyzed by the CCK-8 assay. (B-J') in vitro TUNEL analysis of mammary stromal fibroblasts from 7-week mammary glands. (B, B') positive control of TUNEL analysis. (C-J') TUNEL analysis between control (**C, E, G, I**) and mutant (**D, F, H, J**) subgroups, including MSF-1 (**C-D'**), MSF-2 (**E-F'**), MSF-3* (**G-H'**), and MSF-4 (**I-J'**). The positive control was prepared using the manufacturer's standard protocol, where DNase I was used to create DNA breaks in cells, which were then detected by the TUNEL kit to show that the experimental procedure was correctly performed. Note, consistent with the literature, few dead cells were observed in control and mutant fibroblasts during an active stage of proliferation.

Figure S6. Bioinformatic analysis of MSF subgroups.

(**A**, **B**) Gene Ontology (**A**) and KEGG (**B**) analyses of each of the MSF-1 to MSF-4 subgroups of cells.

Figure S7. Gating strategy for sorting MSF subgroups.

(**A, B**) Gating strategy for control and KO. (C) Western blotting analysis of the MSF-1, 2, and 4 subgroups using CD26, CD36, and CD142 antibodies. GAPDH was used as an internal control.

Figure S8. MSF-2 subgroup consists of activated fibroblasts.

(**A-B'''**) Confocal images of immunofluorescence of PDGFR (**A**, **B**), CD36(**K**, **L**), DAPI and overlay around control (**A-A'''**) and Spry-TKO (**B-B'''**) mammary ducts using frozen sections of mammary glands at the 7-week stage. No CD36 positive "activated fibroblasts" were detected in either control or Spry-TKO glands. Scale bars: 20 μm.

Supplementary Figure 1. Loss of *Spry1*, *Spry2*, and *Spry4* in mammary stromal fibroblasts causes accelerated epithelial branching .

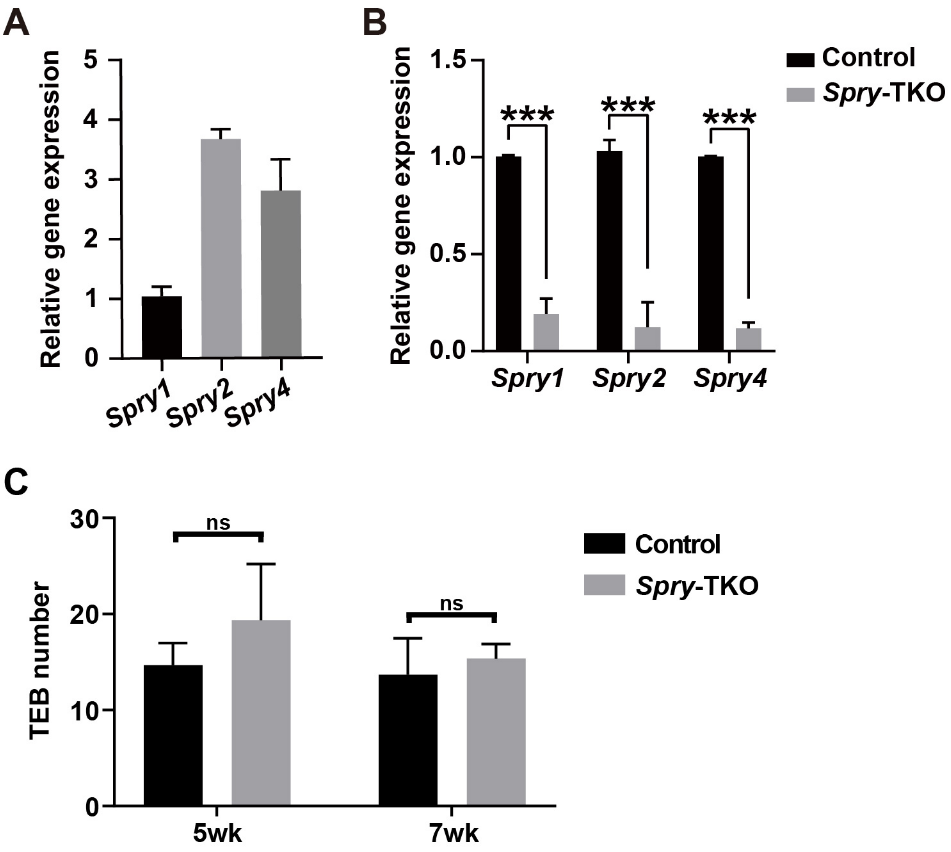

Supplementary Figure 2. Multiple RTK signaling pathways in the fibroblasts are sensitized due to the loss of *Spry1*, *Spry2*, and *Spry4*.

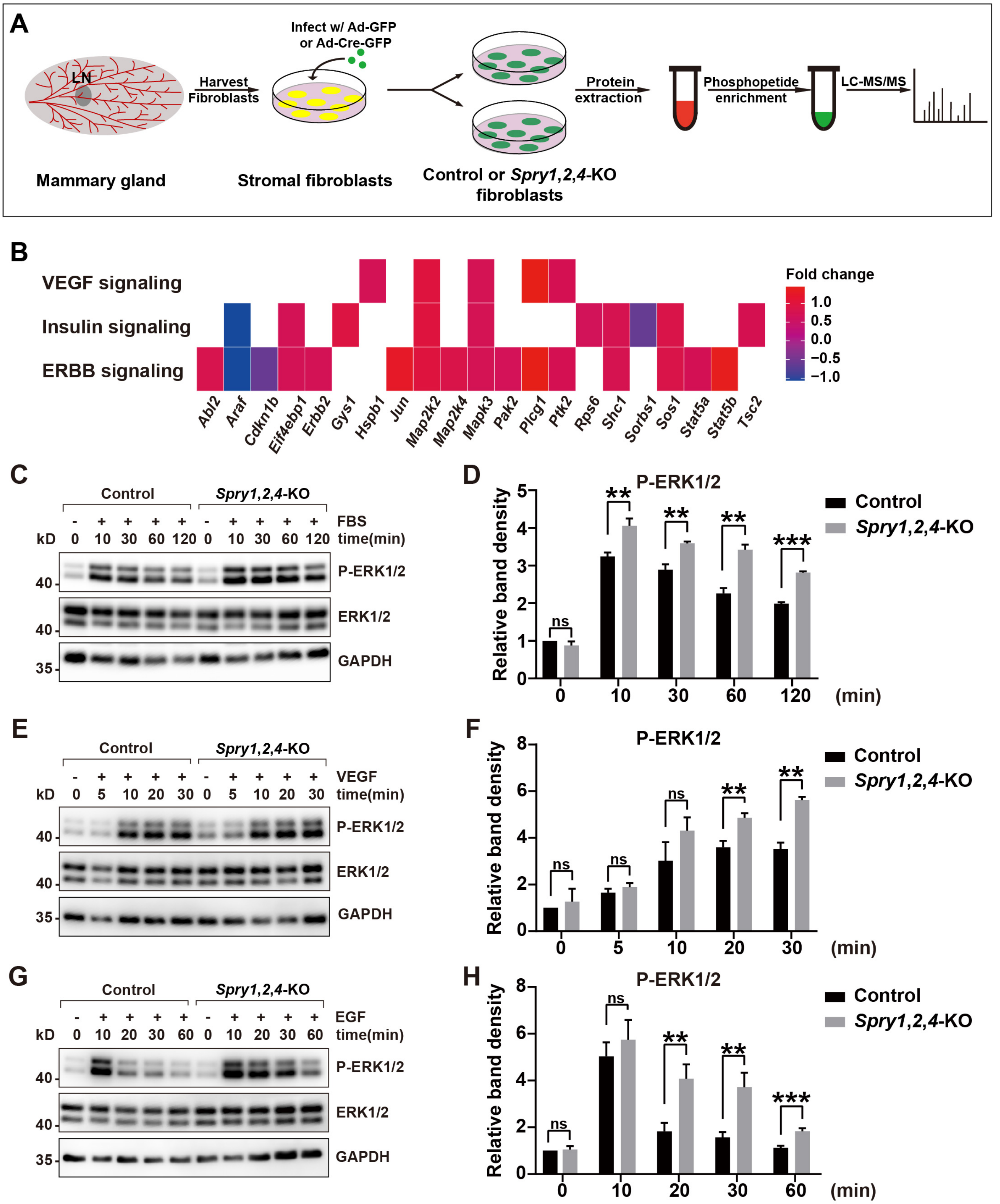

Supplementary Figure 3. Bioinformatic analysis of Cbl and Tgfb1 mRNA expression.

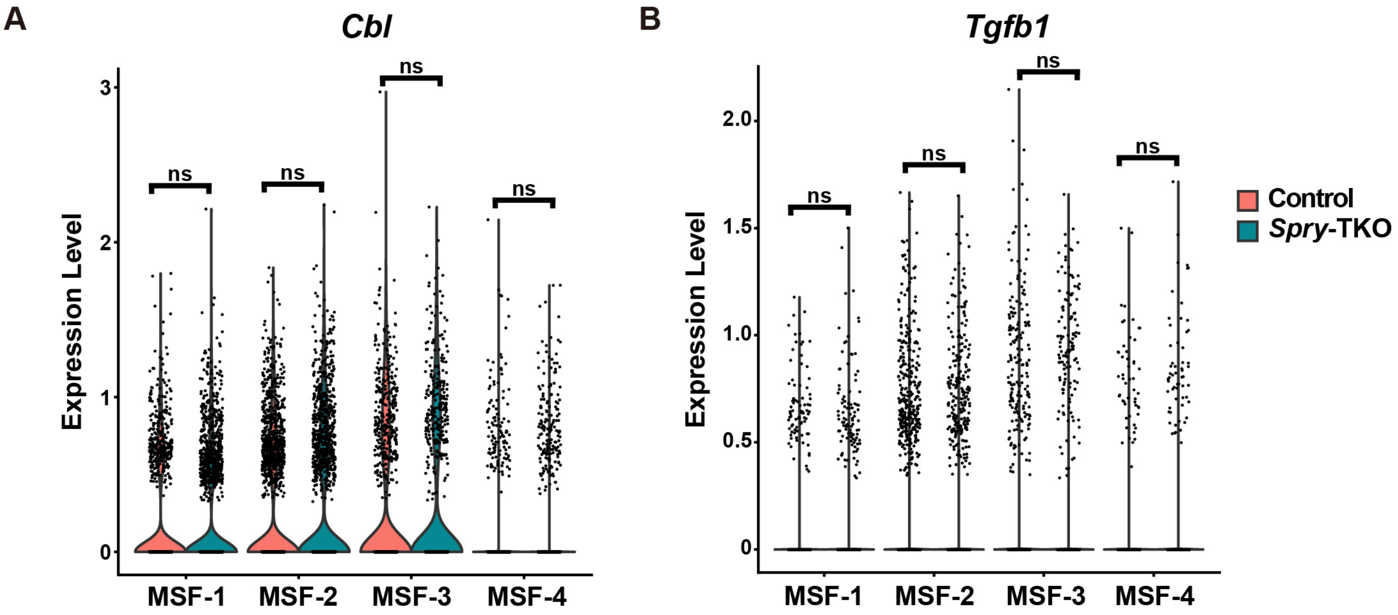

Supplementary Figure 4. MSF-2 subgroup is derived from three independent fibroblast lineages.

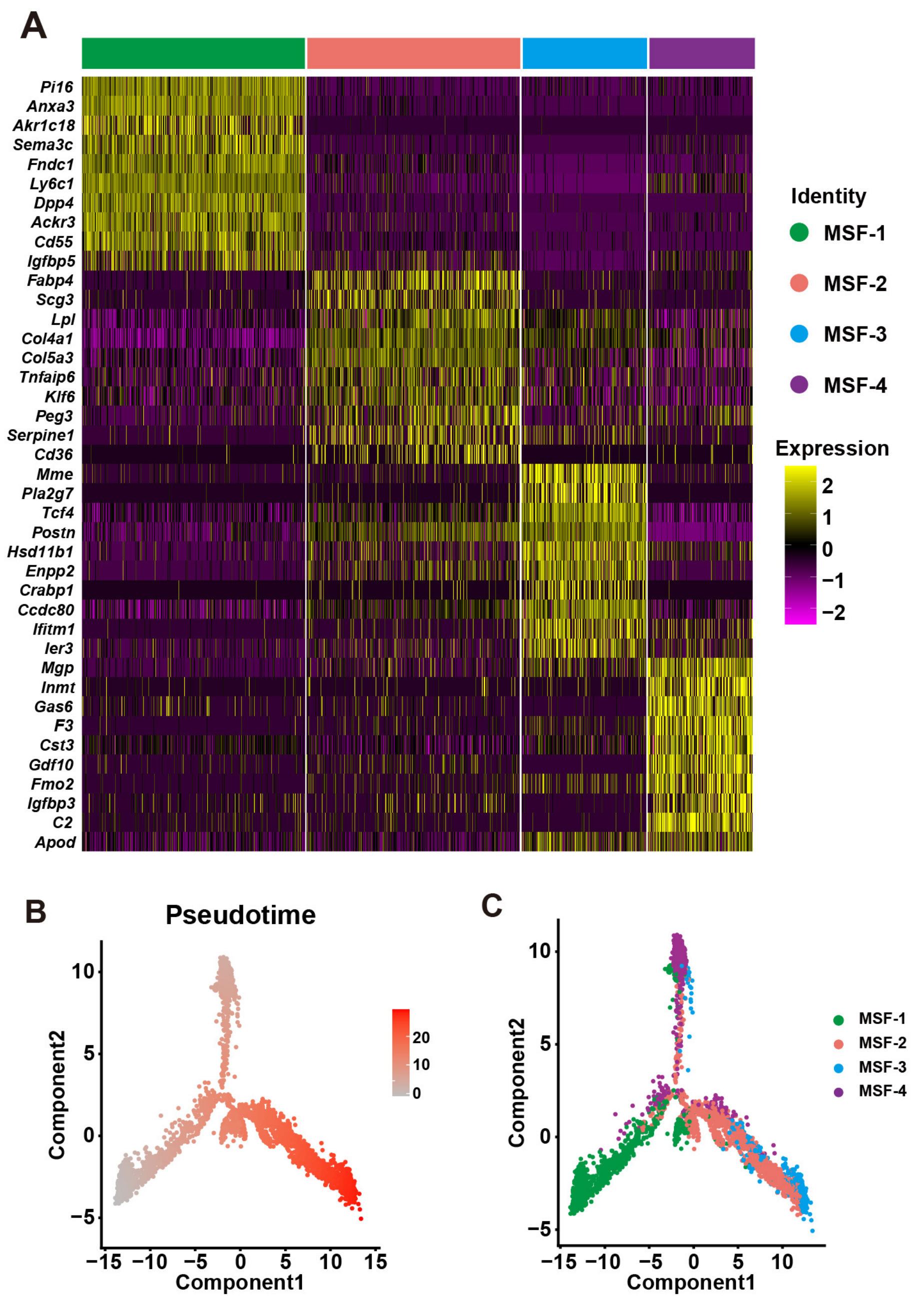

Supplementary Figure 5. Cell proliferation and TUNEL analysis of mammary stromal fibroblasts.

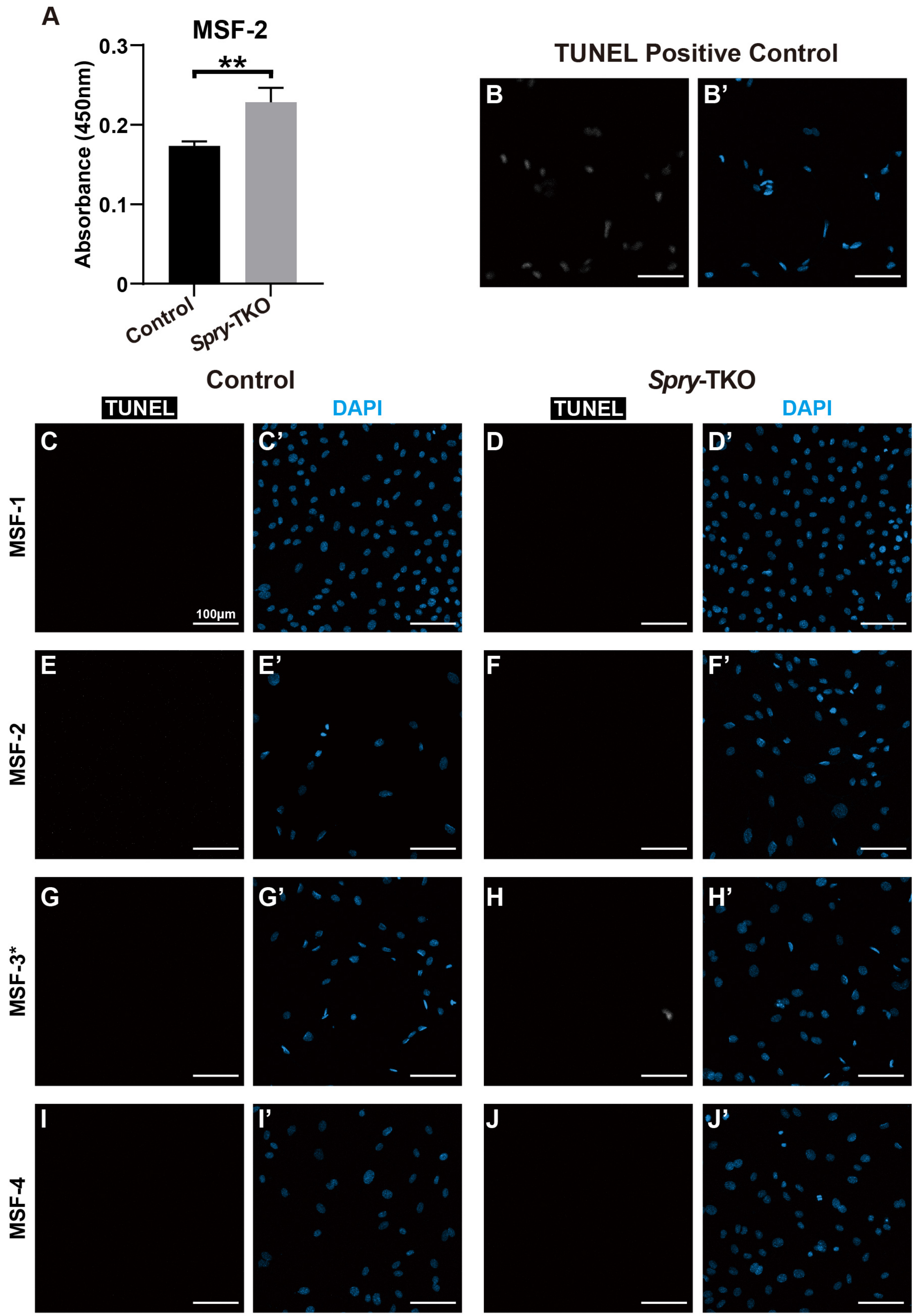

Supplementary Figure 6. Bioinformatic analysis of MSF subgroups.

A. GO analysis

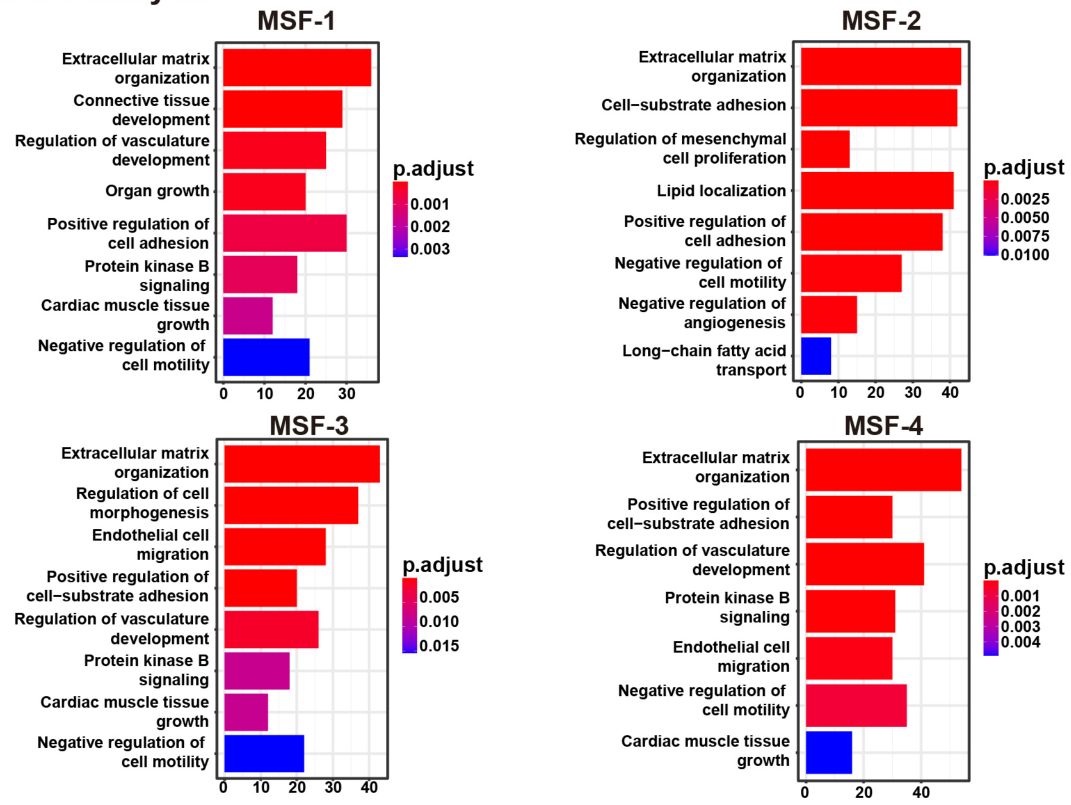

B. KEGG analysis

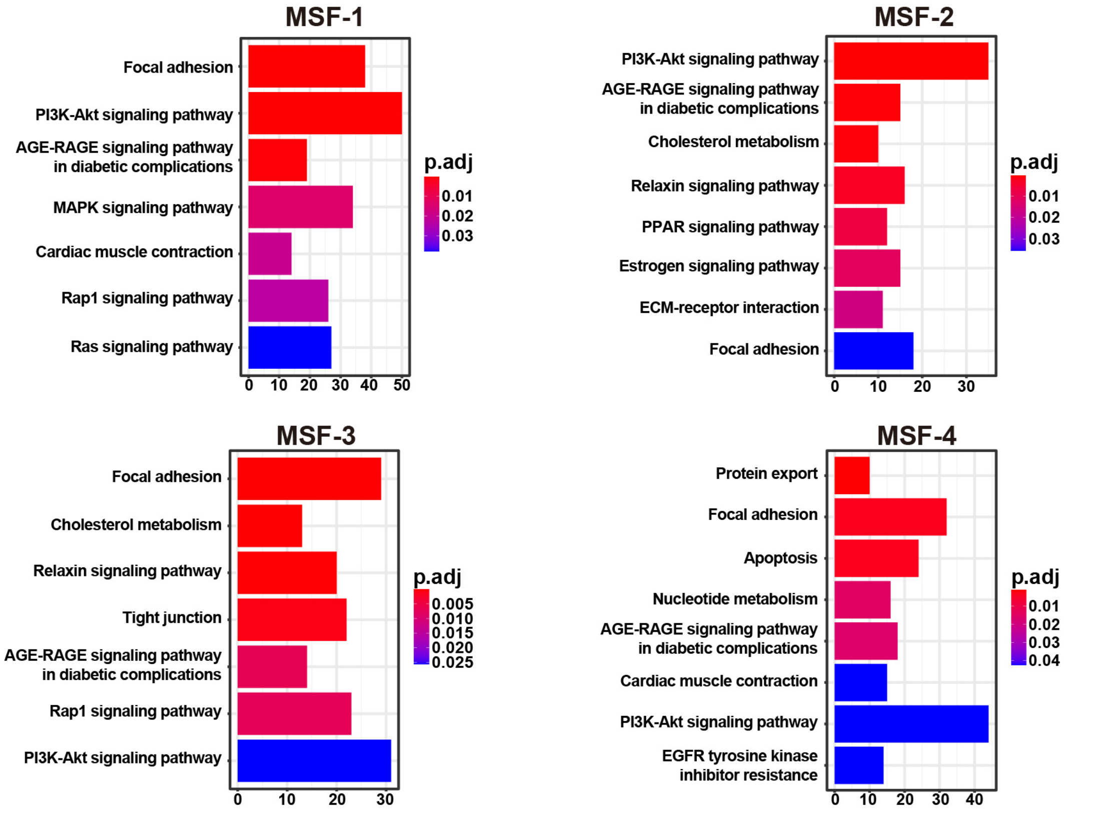

Supplementary Figure 7. Gating strategy for sorting MSF subgroups.

A

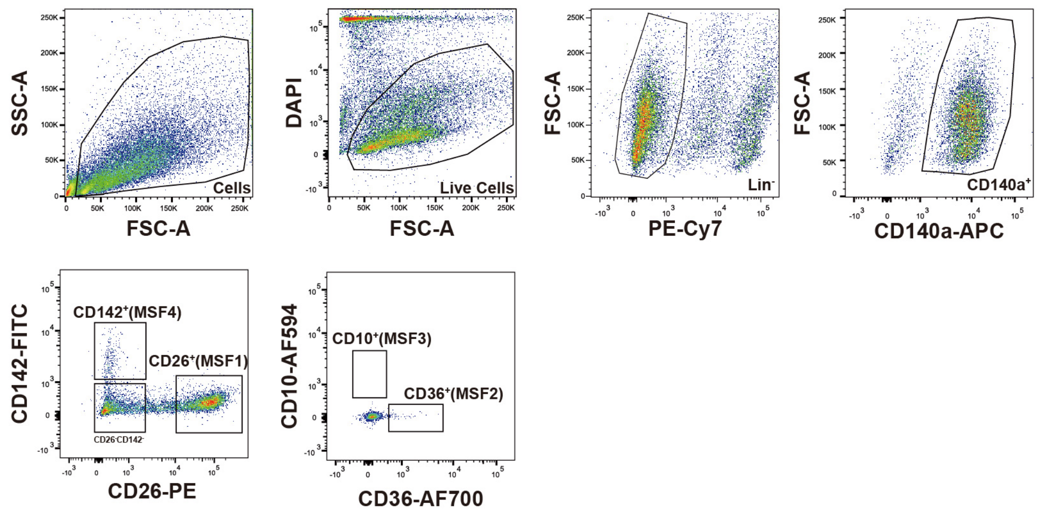

B

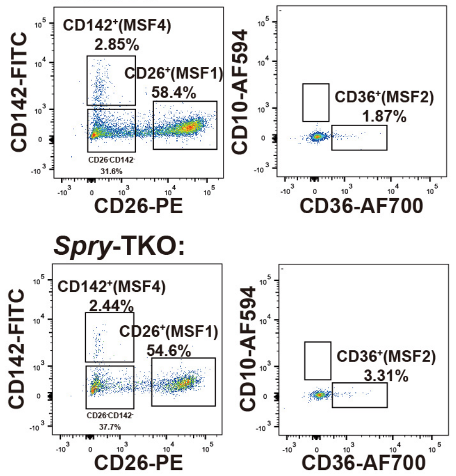

C

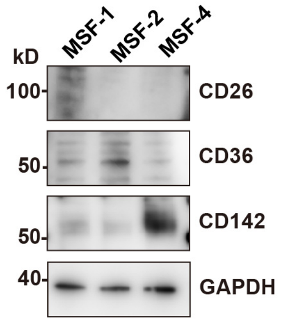

Supplementary Figure 8. **MSF-2 subgroup consists of activated fibroblasts.**

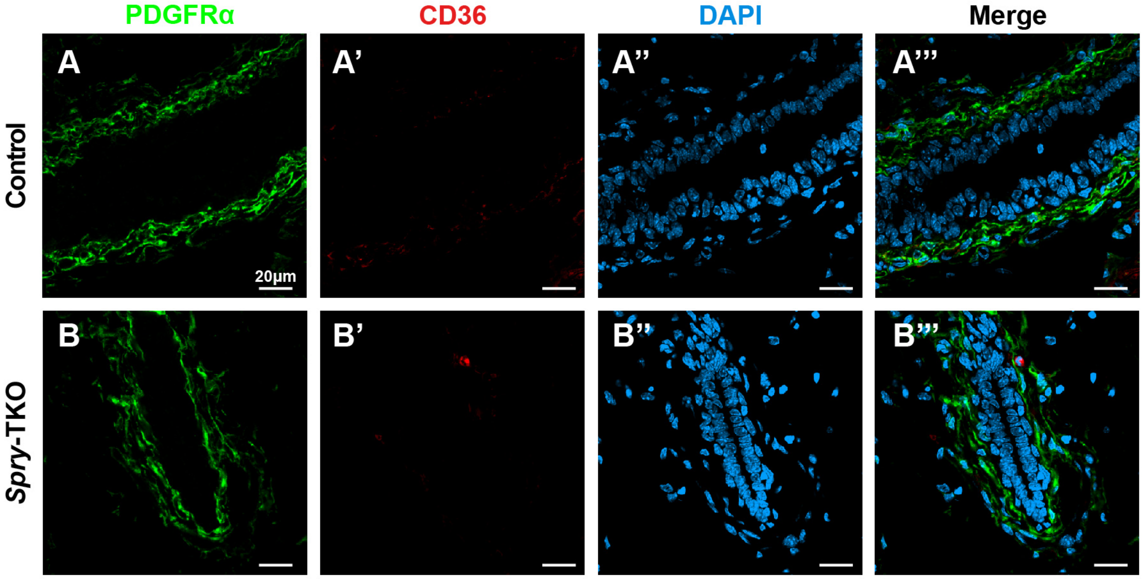

Supplement: Supplementary file 3 — Supplementary Figures and Legends [file 41419_2024_6637_MOESM3_ESM.pdf]
